# Temperature-Dependent tRNA Modifications in Bacillales

**DOI:** 10.3390/ijms25168823

**Published:** 2024-08-13

**Authors:** Anne Hoffmann, Christian Lorenz, Jörg Fallmann, Philippe Wolff, Antony Lechner, Heike Betat, Mario Mörl, Peter F. Stadler

**Affiliations:** 1 Helmholtz Institute for Metabolic, Obesity and Vascular Research, Helmholtz Zentrum München of the University of Leipzig and University Hospital Leipzig, Philipp-Rosenthal-Str. 27, D-04103 Leipzig, Germany; anne.hoffmann@helmholtz-munich.de; 2 Bioinformatics Group, Department of Computer Science, and Interdisciplinary Center for Bioinformatics, Härtelstraße 16–18, D-04107 Leipzig, Germany; fall@bioinf.uni-leipzig.de; 3 Institute for Biochemistry, Leipzig University, Brüderstraße 34, D-04103 Leipzig, Germanyheike.betat@uni-leipzig.de (H.B.); mario.moerl@uni-leipzig.de (M.M.); 4 Architecture et Réactivité de l’ARN, Institut de Biologie Moléculaire et Cellulaire du CNRS, Université de Strasbourg, F-67084 Strasbourg, France; p.wolff@unistra.fr (P.W.); lechnera@igbmc.fr (A.L.); 5 German Center for Integrative Biodiversity Research (iDiv) Halle-Jena-Leipzig, Competence Center for Scalable Data Services and Solutions and Leipzig Research Center for Civilization Diseases, University Leipzig, Puschstrasse 4, D-04103 Leipzig, Germany; 6 Max Planck Institute for Mathematics in the Sciences, Inselstraße 22, D-04103 Leipzig, Germany; 7 Institute for Theoretical Chemistry, University of Vienna, Währingerstrasse 17, A-1090 Wien, Austria; 8 Facultad de Ciencias, Universidad National de Colombia, Bogotá CO-111321, Colombia; 9 Santa Fe Institute, 1399 Hyde Park Road, Santa Fe, NM 87501, USA

**Keywords:** tRNA, RNA modification, bacteria, RNA sequencing, thermal adaption

## Abstract

Transfer RNA (tRNA) modifications are essential for the temperature adaptation of thermophilic and psychrophilic organisms as they control the rigidity and flexibility of transcripts. To further understand how specific tRNA modifications are adjusted to maintain functionality in response to temperature fluctuations, we investigated whether tRNA modifications represent an adaptation of bacteria to different growth temperatures (minimal, optimal, and maximal), focusing on closely related psychrophilic (*P. halocryophilus* and *E. sibiricum*), mesophilic (*B. subtilis*), and thermophilic (*G. stearothermophilus*) Bacillales. Utilizing an RNA sequencing approach combined with chemical pre-treatment of tRNA samples, we systematically profiled dihydrouridine (D), 4-thiouridine (s^4^U), 7-methyl-guanosine (m^7^G), and pseudouridine (Ψ) modifications at single-nucleotide resolution. Despite their close relationship, each bacterium exhibited a unique tRNA modification profile. Our findings revealed increased tRNA modifications in the thermophilic bacterium at its optimal growth temperature, particularly showing elevated levels of s^4^U8 and Ψ55 modifications compared to non-thermophilic bacteria, indicating a temperature-dependent regulation that may contribute to thermotolerance. Furthermore, we observed higher levels of D modifications in psychrophilic and mesophilic bacteria, indicating an adaptive strategy for cold environments by enhancing local flexibility in tRNAs. Our method demonstrated high effectiveness in identifying tRNA modifications compared to an established tool, highlighting its potential for precise tRNA profiling studies.

## 1. Introduction

More than 100 chemical modifications of the four canonical nucleotides, adenosine (A), cytosine (C), guanosine (G), and uracil (U), have been identified in RNA molecules. All modifications are posttranscriptionally incorporated by a diverse set of chemical reactions including deaminations, isomerizations, glycosylations, thiolations, methylations, and transglycosylations [1] and affect not only A, C, G, and U but also create hypermodifications such as queuosine or wybutosine from already modified nucleotides [1,2]. The most diverse collection of modifications has been found in ribosomal RNAs (rRNAs) and tRNAs [3].

With the advent of cost-effective high-throughput sequencing, it has become possible to assess modified nucleotides on genome- and transcriptome-wide scales. The common basis of the broad class of techniques is the use of reverse transcription (RT) that terminates just before certain modifications (e.g., for 1-methyladenosine (m^1^A) and 1-methylguanosine (m^1^G)) and/or misincorporates nucleotides at very high rates when encountering other modifications. The resulting complementary DNA (cDNA) is then subjected to high-throughput sequencing, mapped to a reference genome, and computationally analyzed for strong arrests of primer extension (RT-stops) and position-specific mismatch profiles [4,5,6,7,8]. These events constitute a RT signature that varies depending on the modification type and the used reverse transcriptase [4,5,6,7,8]. Lysidine, for example, is found at the anticodon position 34 of tRNA^Ile^ in bacteria [9,10]. In this modification, cytidine is replaced by a lysine moiety that substitutes the carbonyl group at the Watson–Crick edge. This structural change alters the base-pairing properties with uracil, making this position identifiable in the cDNA sequence profile as a C-to-U substitution in the mapping profile. Another example is inosine, which can pair with cytidine, resulting in a modified hydrogen bonding pattern. This modification can manifest in the sequencing data as an A-to-G mismatch, providing valuable insights into RNA editing events [8].

While some modifications directly affect reverse transcriptase activity or fidelity, other modifications require a preceding chemical treatment to become visible. The treatments can lead to a conversion of the modified nucleotide or induce a block of the primer extension by the reverse transcriptase. As a result, the read-out of the chemically treated nucleotide is different compared to the untreated control. Thus, these modifications can be directly detected by comparing treated versus untreated samples. For example, Ψ becomes detectable after treatment with carbodiimides that acylate one of the nitrogen positions of the base, resulting in Ψ-1-cyclohexyl-(2-morpholinoethyl)carbodiimide (CMC) adducts that block RT [11,12,13]. The saturated pyrimidine ring of D can be opened by sodium borohydride (NaBH_4_) or under alkaline conditions. As a result, base pairing is impeded, leading to straightforward detection by RT signatures. In addition, m^7^G reduction by NaBH_4_ leads to a depurination in RNA followed by the cleavage of the RNA chain by β-elimination [14].

Mainly, tRNA modifications act as checkpoints for tRNA identity as well as integrity, regulate translation, modulate the structural stability, and ensure the correct folding of the tRNA molecule [15]. They are further involved in the structural fine-tuning of local elements and rapidly adapt tRNAs to environmental changes, such as stress and temperature [16,17]. Most chemical modifications modulate the tRNA structure in terms of stability and flexibility [18]. m^1^A at position 9 in the human mitochondrial tRNA^Lys^, for example, disrupts a Watson–Crick base pair (A9-U64) that stabilizes a non-functional rod-like structure and forces the tRNA to adopt the typical cloverleaf secondary structure and the L-shaped three-dimensional functional conformation [19]. Other modifications in the D- and TΨC- loop, carrying D and Ψ, respectively, are involved in tertiary base pairs and enhance the folding of the transcript into the L-shape [20,21]. Pseudouridine, in particular, contributes to the overall stability of tRNA structures with the help of water-mediated bridging interactions between modified bases and the RNA backbone [22,23]. Dihydrouridine, on the other hand, frequently found in the D-arm, is the only non-planar base and thus reduces stacking interactions, conferring a certain flexibility to tRNAs [24].

Initial studies indicate that the control of flexibility versus rigidity indeed plays a role in the temperature adaptation of microorganisms. Accordingly, high levels of D are found in psychrophilic organisms, where flexibility in macromolecules is interpreted as a frequent strategy for cold adaptation [25,26]. In psychrophilic Archaea, tRNAs contain a higher amount of D modifications than their thermophilic counterparts [1,27]. Pseudouridine levels in bacteria can also change as a response to growth temperature [28,29]. However, these analyses investigated the overall change of certain modifications but did not analyze changes at the resolution of individual tRNA sequences. Hence, it is an interesting question as to how modification levels at individual positions in the tRNAs are adjusted in response to temperature changes to keep these molecules functional.

To investigate a possible correlation between temperature and posttranscriptional modifications in bacterial tRNAs, four closely related Bacillales with different growth temperature profiles (*Bacillus subtilis* (mesophilic), *Exiguobacterium sibiricum*, *Planococcus halocryophilus* (both psychrophilic), and *Geobacillus stearothermophilus* (thermophilic)) were used as model organisms. These strains were cultured at temperature ranges from 10 to 70 °C, including the individual optimal growth temperatures as well as the minimum and maximum temperatures feasible in the laboratory. Together, this allowed a screen for temperature-dependent, short-term changes in the tRNA modification pattern within the organism and long-term evolutionary adaptation to their particular habitat.

## 2. Results

### 2.1. Specific Library Design for tRNA Sequencing and Subsequent Analysis of tRNA Modifications

**Figure 1:**
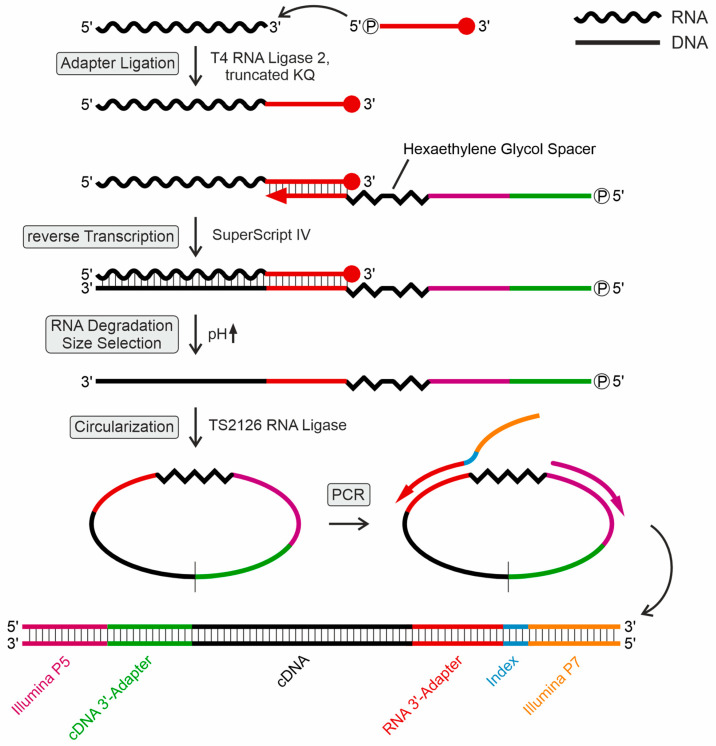
Schematic workflow for library preparation. First, the 3′-protected RNA adapter was adenylated by TS2126 ligase. Subsequently, T4Rnl2KQ was used to ligate the adapter to the 3′-end of transfer RNAs (tRNAs). The adapter introduced the index primer binding site and served as a primer binding site for reverse transcription (RT). The RT primer carries a hexa-ethylene glycol spacer to prevent rolling circle polymerase chain reaction (PCR) amplification. After RT, RNA was degraded and complementary DNA (cDNA) was size-selected in a polyacrylamide gel. cDNA was then circularized and served as a PCR template. The PCR primers introduced Illumina flow cell sequences P5 and P7, along with one of the 25 6-bp i7 indices used for multiplexing samples on the Illumina sequencing platform.

For a systematic detection of dihydrouridine (D), pseudouridine(Ψ), and 7-methylguanosine (m^7^G) modifications in the tRNA mapping profiles, individual tRNA samples were chemically treated to convert these modifications to yield a specific read-out in the subsequent sequencing step compared to untreated control samples. From each bacterial culture, small RNA fractions were prepared by high salt precipitation of the high molecular weight RNA. A quality assessment of the small RNA preparations was performed on a BioAnalyzer 2100 device (Agilent, Santa Clara, CA, USA) as well as on high-resolution denaturing polyacrylamide gels. Subsequently, individual small RNA preparations were treated with sodium borohydride(NaBH_4_) (for D and m^7^G profiling) and 1-cyclohexyl-(2-morpholinoethyl)carbodiimide metho-p-toluene sulfonate (CMCT) (for Ψ identification). Treated as well as untreated samples were then used for library preparation and RNA sequencing (RNA seq) on an Illumina MiSeq device (experimental protocol is illustrated in Figure 1). To this end, a 5′-phosphorylated DNA adapter was ligated to the 3′OH ends of the small RNAs. The 3′-end of this adapter was amino-C7-blocked to avoid concatemer formation. To ensure efficient ligation, the adapter was used in a two-fold excess compared to the small RNA preparation.

**Figure 2:**
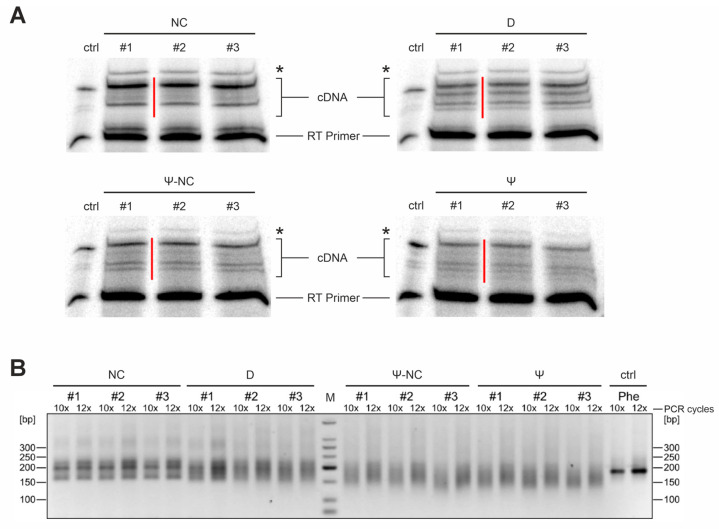
Library preparation for Illumina RNA sequencing. Libraries for *B. subtilis* tRNAs at 20 °C are shown as a representative example. (**A**) cDNA synthesis of untreated (negative control, NC), sodium borohydride (NaBH_4_)-treated (dihydrouridine detection, D), 1-cyclohexyl-(2-morpholinoethyl)carbodiimide metho-p-toluene sulfonate (CMCT)-treated (pseudouridine detection, Ψ), and CMCT control sample (pseudouridine-negative control, Ψ-NC) were separated on preparative denaturing polyacrylamide gels for subsequent isolation. The red bars indicate the cut-out bands. The asterisks indicates the signal for 5S ribosomal RNA (verified by cloning and Sanger sequencing of individual clones). Accordingly, this band was omitted from further analysis. Numbers 1 to 3 represent the three biological replicates. Ctrl, control cDNA synthesis of in vitro transcribed yeast tRNA^Phe^. (**B**) Amplified sequencing libraries of the individual tRNA treatments shown above. PCR cycles (10× or 12×) are indicated. All libraries show the expected smear, while the amplification product of an in vitro transcribed yeast tRNA^Phe^ (control, ctrl, Phe) is represented by a single sharp band.

Next, the ^32^P-5′-labeled reverse transcription (RT) primer (Supplementary Table S1) was hybridized to the small RNA (sRNA)/adapter ligation product. At its 5′-end, this primer carried the complementary DNA (cDNA)-3′-adapter region for subsequent circularization of the cDNA products by TS2126 RNA ligase (CircLigase). Furthermore, it contained the sequence corresponding to the Illumina P5 adapter (Figure 1). For cDNA synthesis, SuperScript IV reverse transcriptase was used, and the resulting radioactively labeled reaction products were separated and visualized on a denaturing 15% polyacrylamide gel (a representative example is shown in Figure 2A). Bands representing cDNA products were cut out, isolated, circularized, and directly used for polymerase chain reaction (PCR) amplification. A single band migrating at a higher position than the tRNA signals was identified by Sanger sequencing after cloning and omitted from further analysis. To avoid saturation effects and an amplification bias, the PCR reaction was limited to 10–12 cycles (Figure 2B, representative example). One PCR primer corresponded to the Illumina P5 flow cell linker sequence and the second one hybridized to the RNA-ligated 3′-adapter and introduced the Illumina sequence (Supplementary Table S1) as well as an index region for identification of the individual library sequences. The indexing allowed for a mixing of the individual amplification products and a combined sequence analysis. A total of 36 libraries per organism were prepared, consisting of three biological replicates, each grown at the three indicated temperatures. For each temperature sample, the following four treatments were performed: untreated sRNA preparation, NaBH_4_ treatment (detection of D, m^7^G), CMCT treatment, and CMCT-negative control (detection of Ψ). Subsequently, the pooled libraries were sequenced on an Illumina MiSeq device.

**Figure 3:**
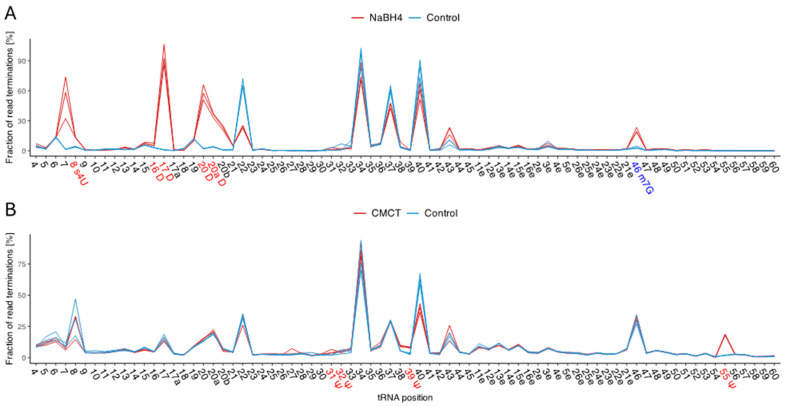
Quantitative representation of the RNA seq data for *B. subtilis* at 20 °C. The percentage of reverse transcriptase stops for each tRNA position is provided. The mean value of each tRNA position across all clusters was utilized. The three replicates for the untreated control samples (blue) and the (**A**) NaBH_4_- and (**B**) CMCT-treated RNA seq data (red) are displayed. Detected modifications are highlighted on the y-axis. High peaks in the treatments and low peaks in the controls indicate an enrichment of RT stops, suggesting modifications. However, modifications occurring in only a few tRNA clusters show a weaker increase in peak height between treatments and controls.

After sample quality assessment and normalization of the resulting sequences, we scanned for primer extension (RT-stop) sites that were significantly enriched (false discovery rate (FDR) adjusted *p* value < 0.01) in the mapping profile of the treated libraries compared to the untreated control sample according to the Poisson distribution. Additionally, we distinguished true modification sites from spurious noise. Thus, significant RT-stop sites with a log_2_ fold change (FC) ≤ 1 were filtered out, since we assumed that the modified sites of D, m^7^G, and Ψ showed a higher effect size due to the treatment than that from noise. Further, low read coverage at specific tRNA positions may result in overestimation of the ratio of RT-stops to the total number of reads. According to our observations, treatment-based RT-stop sites have a higher ratio of reads with RT-stops to reads without RT-stops in the corresponding position compared to noise. Therefore, only RT-stop sites with a total number of RT-stops ≥ 20 and a percentage of RT-stops ≥ 2 were considered. The thresholds were chosen to be close to the maximum value of potential true positive sites (TPs) at which known sites are consistently predicted, while keeping the number of false positive sites (FPs) as low as possible. Figure 3 shows example data for *B. subtilis* at 20 °C, illustrating both treatments and the controls for each replicate. The replicates show a consistent pattern for each condition, supporting the quality and reproducibility of the data.

**Figure 4:**
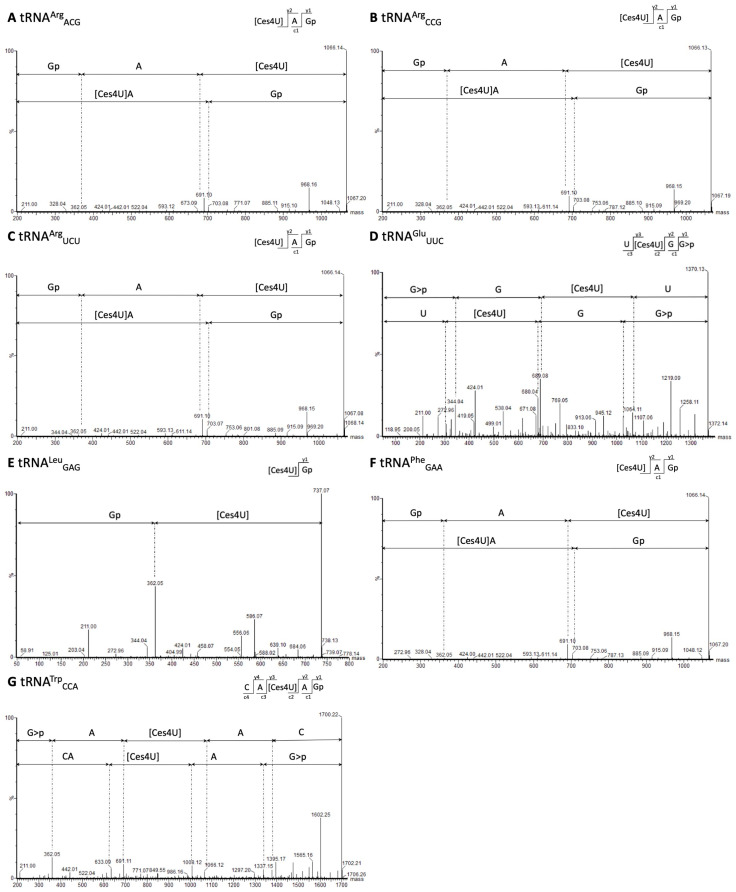
MS/MS sequencing spectra containing cyanoethylated 4-thiouridine (Ces^4^U) at tRNA position U8 of *G. stearothermophilus*. (**A**) MS/MS spectrum [Ces^4^U]AGp of tRNA^Arg^_ACG_ after RNase T_1_ digestion (*m*/*z* 1066.14 z = 1-). (**B**) MS/MS spectrum [Ces^4^U]AGp of tRNA^Arg^_CCG_ after RNase T_1_ digestion (*m*/*z* 1066.13 z = 1-). (**C**) MS/MS spectrum [Ces^4^U]AGp of tRNA^Arg^_UCU_ after RNase T_1_ digestion (*m*/*z* 1066.14 z = 1-). (**D**) MS/MS spectrum U[Ces^4^U]GG>p of tRNA^Glu^_UUC_ after RNase U_2_ digestion (*m*/*z* 684,57 z = 2-). (**E**) MS/MS spectrum [Ces^4^U]Gp of tRNA^Leu^_GAG_ after RNase T_1_ digestion (*m*/*z* 737,07 z = 1-). (**F**) MS/MS spectrum [Ces^4^U]AGp of tRNA^Phe^_GAA_ after RNase T_1_ digestion (*m*/*z* 1066.13 z = 1-). (**G**) MS/MS spectrum CA[Ces^4^U]AGp of tRNA^Trp^_CCA_ after RNase T_1_ digestion (*m*/*z* 849.61 z = 2-).

To classify FP and TP sites of potential D, m^7^G, and Ψ modifications, knowledge from the RNA modification database MODOMICS [30] and the scientific literature [3,5,31,32,33,34,35,36,37] was utilized regarding the known bacterial tRNA positions where these modifications are present. The MODOMICS database (https://genesilico.pl/modomics/, accessed on 3 January 2024) contains 383 bacterial tRNA sequences, with only four tRNA sequences for *G. stearothermophilus* and 25 for *B. subtilis* but none for *P. halocryophilus* and *E. sibiricum*. As only limited information is known about tRNA modifications in these bacteria, tRNA modifications from other bacterial species were also used as a reference. Thus, for the NaBH_4_-treated RNA seq data, RT-stop sites for tRNA U positions 16, 17, 20, 20a, 20b, 21, and 47 were counted as TP D modifications and signals at G46 as TP m^7^G modifications. Upon first inspection, we observed RT-enriched sites at position U8 for several tRNAs, which, due to their strong enrichment, did not appear to arise from noise. However, D modifications are not known to occur at U8 in bacteria; instead, 4-thiouridine (s^4^U) modifications are known [30,38,39,40]. To determine whether the modifications were D or s^4^U, we conducted tandem mass spectrometry (MS/MS) analysis in *G. stearothermophilus* (55 °C growth temperature). We successfully identified s^4^U modifications in tRNA^Arg^_ACG_, tRNA^Arg^_CCG_, tRNA^Arg^_UCU_, tRNA^Glu^_UUC_, tRNA^Leu^_GAG_, tRNA^Phe^_GAA_, and tRNA^Trp^_CCA_ (Figure 4), where we also detected significant RT-stop enrichments in their mapping profile (Supplementary Figure S6). Moreover, based on MS/MS analyses, we identified s^4^U modifications for tRNA^Cys^_GCA_, tRNA^Gly^_CCC_, tRNA^Leu^_UAG_, tRNA^Ser^_UGA_, and tRNA^Tyr^_GAU_ for which we could not find significantly enriched RT-stops (Supplementary Figure S1). Based on this validation through an independent approach, we assume detecting s^4^U at position U8 using our NaBH_4_-treated RNA seq data and have classified these sites as TPs.

For the CMCT-treated RNA seq data, Ψ modifications are known to occur in bacteria at U positions 13, 31, 32, 38, 39, 40, 55, and 65. Upon initial inspection, we observed RT-enriched signals at position U60 in several tRNAs (Supplementary Figures S7–S10), where Ψ modifications were previously unknown in bacteria. Through independent MS/MS analysis in *G. stearothermophilus* (grown at 55 °C), we confirmed that these were Ψ60 modifications (Supplementary Figure S2). RT-stops at these positions were counted as TPs Ψ modifications. FPs were counted as type I errors, where all RT-stop sites for Ts and Gs (Gs only in the NaBH_4_-treated data) occur at tRNA positions where no modifications are previously known. Type II FPs were counted as all RT-stop sites at C, A, or G positions (Gs only in the CMCT-treated data). In summary, this systematic approach allowed us to accurately detect and classify D, Ψ, s^4^U, and m^7^G modifications in tRNAs while minimizing false positive signals.

### 2.2. Variations in tRNA Modification Profiles among Closely Related Bacillales

**Figure 5:**
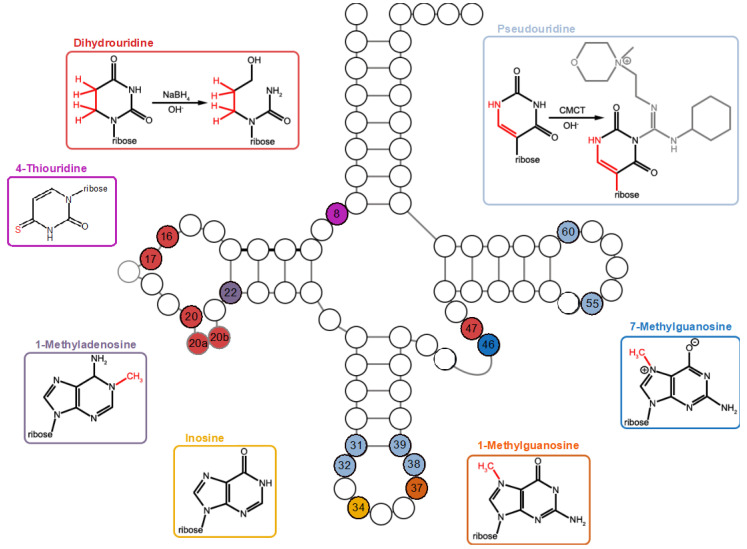
tRNA modification patterns of *P. halocryophilus*, *E. sibiricum*, *B. subtilis*, and *G. stearothermophilus*. Individual modifications were detected either through accumulations of base-calling errors in the mapping profile or by evaluating read-end distributions of chemically treated RNA seq libraries generated by induced primer extension (RT-stops). Each of the tRNA modifications illustrated was identified within our analysis in at least one tRNA type of each bacterium, except for 1-methylguanosine (m^1^G) (at G37, exclusively found in *P. halocryophilus*; dihydrouridine at U20b, present only in *E. sibiricum*; and Ψ38, detected solely in *P. halocryophilus* and *E. sibiricum*. The modified tRNA positions are color-coded to their corresponding modification.

To investigate the general modification profiles of the four closely related Bacillales, *Planococcus halocryophilus*, *Exiguobacterium sibiricum*, *Bacillus subtilis*, and *Geobacillus stearothermophilus*, the bacteria were cultivated at their optimal growth temperatures. These temperatures are 20 °C for *P. halocryophilus* and *E. sibiricum*, 30 °C for *B. subtilis*, and 55 °C for *G. stearothermophilus*. A summary of all profiled modified tRNA positions in the four bacteria is illustrated in Figure 5.

Only the chemically untreated RNA seq data were utilized to identify tRNA modification patterns in the mapping profile that are clearly visible as accumulations of base misincorporations (Supplementary Table S2). In all organisms examined, more than 99% of the reads that could be assigned to tRNA^Arg^_ACG_ clusters have a guanosine instead of the genomically encoded adenosine at tRNA position 34 in the anticodon loop. This is a strong indication of enzymatic deamination of A34 to (inosine) I34 [5,7,8]. The altered base pairing properties of inosine lead to preferential base pairing with cytosine during cDNA synthesis, resulting in identification as guanosine in the mapping profile of RNA seq data. In the D-stem of several tRNAs of all four bacteria, a more extensive pattern of misincorporation was detected at position A22. In these modified tRNAs, the genomically encoded base is adenosine, which is typically identified as guanosine or thymine in the read pattern, indicating a 1-methyladenosine (m^1^A)22 modification [5,6,7,14]. Only in the tRNA^Arg^_CCG_ of *P. halocryophilus*, was a mismatch pattern found at position G37 of the tRNA anticodon. The underlying modification indicates a 1-methylguanosine (m^1^G)37 [5,14].

Analyzing the chemically treated RNA seq data revealed numerous strong RT-stop sites (NaBH_4_: Supplementary Table S3; CMCT: Supplementary Table S4). For a comprehensive overview of all identified s^4^U, D, and m^7^G modifications, as well as type I FPs for each tRNA cluster and bacterium, please refer to Supplementary Figures S3–S6. Using the NaBH_4_-treated RNA seq data, several tRNA genes show s^4^U8, D16, D17, D20, D20a, D20b, D47, and m^7^G46 modifications in each bacterium except for *E. sibiricum*, which lacks s^4^U and D20b modifications and *B. subtilis* lacking D47. Additionally, all four bacteria exhibit strong FPs RT-stop signals at G45. These RT-stop sites only occur if m^7^G46 is present.

In the CMCT-treated RNA seq data at the optimal growth temperature, all four bacteria show Ψ31, Ψ39, and Ψ55 modifications (refer to the overview of detected Ψ modifications and type I FPs in Supplementary Figures S7–S10). However, Ψ32 was not found in *P. halocryophilus*, Ψ38 is only present in the two psychrophilic bacteria, and Ψ60 is not present in *E. sibiricum*. Since Ψ32 and Ψ60 are present at the other growth temperatures considered, it can be assumed that these modifications occur in the respective bacteria.

Additionally, strong RT-stop sites at position U54 are present in many tRNAs across all bacteria. In bacteria, only 5-methyluridine (m^5^U) modifications are known at this position. Unfortunately, a CMCT treatment is not suited to detect such modifications, making it difficult to definitively determine whether it is a Ψ or another modification without further validation. Other FPs in both treatments seem to occur randomly and vary among bacteria.

Even though most of the profiled modifications occur in the tRNA pool of the four bacteria, they vary in the number of affected amino acid families (grouped by the corresponding encoded anticodon triplets) for each modified tRNA position. For example, while m^1^A22 modifications were found in the same tRNAs (tRNA^Tyr^_GUA_, tRNA^His^_GUG_, tRNA^Glu^_UUC_, tRNA^Glu^_UUG_, tRNA^Cys^_GCA,_ tRNA^Leu^_UAA_, tRNA^Leu^_GAG,_ tRNA^Leu^_CAA_, and tRNA^Ser^_GCU_) in all four bacteria, m^1^A22 modifications in other tRNA^Ser^ and tRNA^Leu^ molecules occur specifically for each bacterium, depending on the encoded anticodon (Supplementary Figure S11).

### 2.3. Specific tRNA Modifications Are Temperature-Dependent

To investigate the tRNA-dependent temperature adaptation mechanisms of closely related psychrophilic (*P. halocryophilus* and *E. sibiricum*), mesophilic (*B. subtilis*), and thermophilic (*G. stearothermophilus*) Bacillales, different individual temperatures between 10 and 70 °C were cultivated. In detail, this comprises the optimal growth temperature, as well as (as far as feasible in the laboratory) the minimum and maximum temperatures: *P. halocryophilus* (10 °C, 20 °C, and 30 °C), *E. sibiricum* (10 °C, 20 °C, and 30 °C), *B. subtilis* (20 °C, 30 °C, and 37 °C), and *G. stearothermophilus* (40 °C, 55 °C, and 70 °C).

Regarding the modifications identified through base misincorporations in the chemically untreated RNA seq data, no strong difference was observed in the fraction of base mismatches for these specific modifications across the investigated bacteria at their respective specific growth temperatures (Supplementary Figure S12), suggesting no stronger temperature dependence of the m^1^A22, I34, and m^1^G37 modifications.

Analysis of D, m^7^G, and Ψ tRNA modifications revealed consistent patterns in *P. halocryophilus*, *E. sibiricum*, and *B. subtilis* within the different temperatures assessed, with similar levels of modified tRNA clusters observed for modifications such as D17, D20a, Ψ39, and m^7^G46 (Table 1). Interestingly, Ψ38 was uniquely identified in the two psychrophilic bacteria. Notably, a distinct trend was observed in *G. stearothermophilus*, where the number of modified tRNAs at specific positions increased with rising temperatures (Table 1). For instance, the abundance of modifications at D17 (40 °C: 1; 55 °C: 12; 70 °C: 13), D20 (40 °C: 6; 55 °C: 18; 70 °C: 19), and Ψ55 (40 °C: 9; 55 °C: 21; 70 °C: 29) exhibited a clear temperature-dependent increase. In contrast, modifications like Ψ32 and m^7^G46 remained relatively stable across temperature variations in *G. stearothermophilus*. Furthermore, a striking observation was the significantly higher occurrence of s^4^U8 modifications in *G. stearothermophilus* (>24) compared to other psychrophilic and mesophilic bacteria (≤4).

### 2.4. Performance Validation

**Figure 6:**
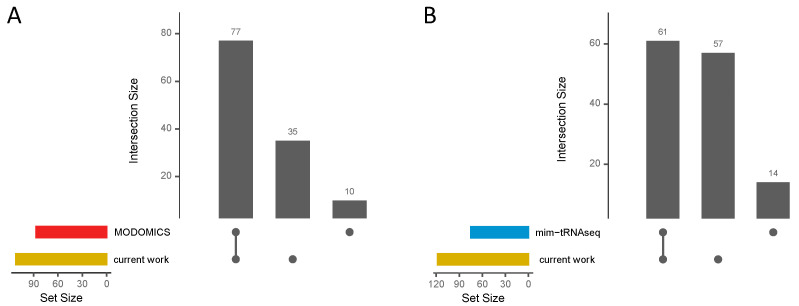
Validation overview. Upset plots, which visually represent the overlaps and unique elements between different datasets, are used to summarize the (**A**) tRNA modifications (m^1^A, m^7^G, s^4^U, D, and Ψ) of specific tRNA genes from *G. stearothermophilus* and *B. subtilis* listed in the MODOMICS database in comparison to the results obtained in this study. (**B**) Upset plot summarizing potential m^7^G, s^4^U, D, and Ψ modifications identified in *B. subtilis* (30 °C) through *mim-tRNAseq* and our current work.

For analysis performance validation, we compared the tRNA modifications available in the MODOMICS database for each listed tRNA gene, including m^1^A, m^7^G, s^4^U, D, and Ψ modifications, with our results for *G. stearothermophilus* and *B. subtilis* (Figure 6A, Supplementary Table S5). For the specific tRNAs available in the database for *G. stearothermophilus* (*n* = 4) and *B. subtilis* (*n* = 21, *n* = 4 not found with *tRNAscan-SE*), we were able to reproduce 89% of the modifications in our analyses, while only 10 out of 87 modifications could not be found. Most of these 10 modifications are present in our data as weak signals; so, they were filtered out by our thresholds as we could not clearly distinguish them from noise. However, we discovered 35 additional modifications for these tRNA genes that are not listed in the database.

Additionally, to verify the validity of our findings using a different analysis method, we used the previously published *mim-tRNAseq* v1.1.6 tool [41] for our *B. subtilis* (30 °C) RNA seq data (Supplementary Table S6). Considering the affected amino acid families grouped by their corresponding encoded anticodon triplets and modified tRNA position (counted uniquely), a total of 61 modifications were identified by both *mim-tRNAseq* and our method (Figure 6B). In addition, 57 modifications were detected only by our analysis, while 14 modifications were found only via *mim-tRNAseq*. The number of type I FPs (*mim-tRNAseq*: 58; own: 63) is approximately the same for both methods (Supplementary Table S6).

In conclusion, our performance validation demonstrated the effectiveness of our method by identifying a large number of previously known modifications for specific tRNA genes. Our comparative study with *mim-tRNAseq* confirmed our results and showed that our method can reliably detect both known and novel modifications.

## 3. Discussion

This study aimed to investigate whether variations in tRNA modifications represent an adaptation of bacteria to different growth temperatures. To address this inquiry, we cultivated four closely related Bacillales species, *P. halocryophilus*, *E. sibiricum*, *B. subtilis*, and *G. stearothermophilus*, under three different temperature conditions (minimal, optimal, and maximal). We combined an RNA seq approach with chemical pre-treatment of the tRNA samples and developed an analysis strategy that allows for systematic profiling of D, s^4^U, and m^7^G (NaBH_4_-treatment) as well as Ψ (CMCT-treatment) modifications in the transcriptome at a single-nucleotide resolution. Based on our statistical quantification of the data, we detected a variety of RT-stop sites that are significantly and strongly enriched in the treated samples compared to the untreated control. A significant challenge was that the tRNA treatments were designed to detect up to three different modifications in the resulting reads. In contrast to gene expression analysis, where statistical robustness and performance can be increased by aggregating reads from an entire gene, measurements of tRNA modifications comprise only single nucleotides [42]. Another challenge was to distinguish true modification sites from noise. Such RT-stop sites occur since the reverse transcriptase is sensitive to certain structural features of the RNA template [43,44] or to other modifications that are not specifically enriched by the treatment. Complex processing of RNA, genomic misalignments of sequencing reads, and technical errors of the sequencing platform contribute to incorrect signals. We assumed that—due to the chemical tRNA treatments—the modified sites result in a considerably higher signal over noise ratio. The use of a specifically adapted FC cutoff is, therefore, suitable. In addition, low read coverage at specific tRNA positions may result in overestimation of the ratio of RT-stops to the total number of reads. In both treatments, these signals are particularly frequent in the TΨC-arm and D-arm, as well as the 5′-acceptor stem of tRNAs. In addition, we observed that the SuperScript IV reverse transcriptase exhibited an increasing tendency to incorporate incorrect bases in the 3′-range and terminate prematurely, leading to background noise in the data. Such signals were filtered out by only considering RT-stop sites that exhibited a minimum number and percentage of RT-stops at the respective position. We were not able to completely remove the background noise without accepting an increased false negative rate by more stringent parameter settings. As expected, approximately equal numbers of type I FPs were observed using the *mim-tRNAseq* tool. However, such FP RT-stop sites showed variability across different bacterial species and growth temperatures, indicating a lack of clear patterns. As ignoring the impact of FP detection likely leads to data misinterpretation, a careful consideration and mitigation of background noise and its incorporation into threshold determination may enhance the accuracy and reliability of tRNA modification profiling studies. To determine the optimal cutoffs, we classified RT-stop sites as TP modifications or FPs based on established information from the RNA modification database MODOMICS [30] and the relevant scientific literature [3,5,31,32,33,34,35,36,37]. It is important to note that this classification was not specific to individual tRNAs, as our focus was on less well-characterized bacteria. Due to the lack of comprehensive characterization of the investigated bacteria, this method is limited in its ability to detect unexpected modifications that may occur during a temperature shift, as the true positive positions of modified tRNA were identified based on existing knowledge. Irrespective of potential false negatives at unexpected sites, our data provide insights into the temperature-dependent changes at those sites where we observe modifications with high confidence.

RNA-sequencing-based detection technologies have become widely adopted for the comprehensive identification of RNA modifications across the transcriptome [8]. These approaches encompass both chemically treated sequencing and direct sequencing methods, which are employed to identify mismatches in the cDNA-to-genome mapping profile. Although direct sequencing methods effectively preserve the status of RNA modifications, they can be complicated by several factors, including RNA secondary structure, single nucleotide polymorphisms, somatic mutations, pseudogenes, and sequencing errors [45,46]. Furthermore, these techniques often necessitate high sequencing depth to accurately detect modifications in low-abundance RNA species [46]. Consequently, our analyses cannot exclude the possibility that we may have detected FPs in untreated RNA seq data.

Upon initial examination of the NaBH_4_-treated RNA seq data, we identified RT-enriched sites at position U8 in several tRNAs, suggesting a specific modification rather than random noise. It is noteworthy that s^4^U modifications are typically associated with U8 in bacteria and archaea [30,38,39,40]. Our mass-spectrometry-based analysis (Figure 4) validated the presence of s^4^U8 modifications in *G. stearothermophilus*. Since we detected both MS/MS signals and enriched RT stops in the same set of 8 tRNAs, we hypothesize that we could also identify s^4^U8 modifications with the NaBH_4_-treated data.

In addition to identifying the general tRNA modification patterns in the investigated bacteria, our study aimed to detect temperature-induced differences in tRNA modifications. The analysis revealed numerous strong RT-stop sites and base misincorporation accumulations in the mapping profiles, indicating the presence of various modifications such as s^4^U, D, m^1^A, m^7^G, m^1^G, I, and Ψ (Figure 5). Despite most of these modifications occurring in the tRNA pool of the four species, the number of each modified tRNA position varied in the individual isoacceptor families. This provided a unique modification profile for each bacterium, even though they are closely related (Supplementary Figure S11). It is important to note that the absence of certain modifications does not necessarily imply their non-existence but could be attributed to weaker expression levels or exclusion by our stringent cutoff criteria. For example, we did not observe strong RT-stops for Ψ38 in the tRNAs of *B. subtilis*, despite previous reports of its presence [14,47]. Further, m^1^G37, involved in frame shift suppression [48], was only found in *P. halocryophilus*, while it is also described in *B. subtilis* [37]. Further validation methods such as deletion of RT-stop signatures via the enzymatic demethylation of m^1^G by AlkB-type enzymes [49] or MS/MS analysis [50,51] could help confirm the presence of these modifications. Similarly, slight variations in the number of modified tRNA clusters within a species across different cultivation temperatures should not be overinterpreted, as the variations may reflect differences in modification intensity rather than distinct modifications. Thus, our cutoff criteria allowed us to infer the presence of tRNA modifications with minimal intensity levels. In addition, strong RT-stop sites at position U54 are observed in numerous tRNAs across the bacterial species. In bacteria, only m^5^U54 (ribothymidine) modifications have been documented [3,30,37,52]. Unfortunately, it is not known whether a CMCT treatment can effectively detect such modifications, posing challenges in definitively classifying the RT-stop sites without further validation. However, the presence of robust RT-stop sites at position U54 in almost all tRNAs aligns with the listing of m^5^U modifications in the MODOMICS database, reinforcing the likelihood that this type of modification has been detected in this context.

The analysis of temperature-dependent tRNA modifications revealed intriguing patterns in psychrophilic and mesophilic bacteria, with consistent trends observed in these organisms. Interestingly, the thermophilic bacterium *G. stearothermophilus* exhibited an increase in the number of tRNAs modified at specific positions as temperatures increased. This observation suggests a temperature-dependent regulation of tRNA modifications. Of particular interest is the significantly higher occurrence of s^4^U8 modifications in *G. stearothermophilus* compared to non-thermophilic bacteria. Previous studies have highlighted the role of s^4^U modifications in stabilizing tRNA structures and influencing translation efficiency [17,53,54,55,56,57]. In particular, the 4-thiolation at U8 is discussed to reinforce a reverse Hoogsteen interaction between this position and A14, increasing the tRNA melting temperature [55,57]. In addition, s^4^U is not a stable, permanent tRNA modification; its presence is regulated by specific RudS-type eraser enzymes [56]. This allows organisms to modulate the levels of s^4^U in their tRNAs, serving as a mechanism for regulating tRNA stability and flexibility. It has also been demonstrated that s^4^U8 is conserved in the tRNAs of both bacteria and archaea, exhibiting photosensitivity and the ability to undergo UV crosslinking with adjacent cytidine residues [54]. Consequently, RudS enzymes may provide protection against such crosslinks—a notion supported by the frequent occurrence of RudS genes in operons that encode UV-induced damage repair enzymes [56]. The increased presence of s^4^U8 in *G. stearothermophilus* could be interpreted as an adaptive strategy to maintain tRNA stability and functionality under extreme high-temperature conditions, potentially serving as a factor for the thermotolerance of this organism. Interestingly, at elevated temperatures of 70 °C, which exceed the optimal growth conditions for *G. stearothermophilus*, there is a slight decrease in s^4^U8 modifications. This reduction may be linked to heat stress impacting tRNA stability and the potential downregulation of the RudS-type eraser enzymes responsible for these modifications. The cell might be attempting to maintain modification levels within an optimal range to prevent overmodification and its associated negative effects. Furthermore, the stability of the s^4^U modification itself could be compromised at such high temperatures, leading to increased degradation. Consequently, the cell may prioritize energy and resources for survival mechanisms rather than sustaining specific tRNA modifications under extreme conditions.

The presence of the Ψ55 modification has been identified as a control mechanism for thermal adaptation of tRNA functionality [17,58]. The presence of Ψ55 limits the incorporation of modifications that provide thermal stability, whereas its absence promotes the addition of such modifications, enhancing thermal stability. Ψ55 was originally identified to stabilize the tRNA structure, as it introduces a further hydrogen-bond donor at the N1 position of the base, where a water molecule can be bound to interact with the neighboring sugar-phosphate backbone [23,57,59]. Yet, Ψ55 can also enhance the flexibility and functionality of tRNAs at lower temperatures. While the investigated psychrophilic and mesophilic Bacillales did not show significant trends for Ψ55 modifications concerning temperature differences, *G. stearothermophilus* exhibited a substantial increase in Ψ55 from 9 modified tRNA clusters at the minimal growth temperature to 29 modifications at the maximal growth temperature. This suggests that Ψ55 may play a crucial role in the thermal adaptation of *G. stearothermophilus* by modulating the stability and flexibility of tRNAs at varying temperatures.

It has been reported that some psychrophilic microorganisms have a higher dihydrouridine content in their tRNAs compared to mesophilic and thermophilic organisms [17,24,26,27,60]. This modification represents the only base that adopts a chair conformation. All other bases in nucleic acids are strictly planar and readily form base-stacking interactions that stabilize and rigidify the surrounding structure. Due to its non-planar structure, dihydrouridine interferes with base stacking and leads to an increased local flexibility in tRNAs, a feature that may represent an important adaptation to cold environments [17,24,26,27,60]. However, we cannot fully confirm this hypothesis, as we do not observe significant differences between psychrophilic and mesophilic bacteria. Nevertheless, the number of D modifications in tRNAs from the psychrophilic and mesophilic bacteria is higher than in the thermophilic bacterium. A possible function for the structural flexibility introduced by D in meso- and thermophilic bacteria is a contribution to the accommodation of the aminoacyl-tRNA bound in the ribosomal A-site, where it undergoes a defined conformational change to allow the peptidyl transfer with the peptidyl-tRNA located in the P-site of the ribosome [57,61].

Only a few tools have already been published for the detection of tRNA modifications using treatment-based RT-stops in sequencing mapping profiles, for example see [41,62]. To compare our findings with those of an already established tool for such analyses, we utilized the *mim-tRNAseq* tool. The *mim-tRNAseq* methodology relies on prior knowledge to classify true positives, like the approach adopted in our analyses. Our method demonstrated high effectiveness, as we were able to identify over 81% of the modifications (grouped by amino acid families and their encoded anticodons) found using *mim-tRNAseq* in *B. subtilis*, along with an additional 57 modifications (Figure 6B). Furthermore, we successfully identified 89% of the specific modifications listed in the MODOMICS database for *G. stearothermophilus* and *B. subtilis*, as well as detecting an additional 35 modifications for specific tRNA genes (Figure 6A). This comprehensive coverage of known modifications highlights the robustness and reliability of the obtained data at a single-base resolution. The ability to detect both previously known and novel modifications underscores the potential of our method for advancing the understanding of tRNA biology.

## 4. Materials and Methods

### 4.1. Cultivation of Bacteria

*B. subtilis* strain *DSM 10*, *E. sibiricum* strain *DSM 17290*, *P. halocryophilus* strain *DSM 24742*, and *G. stearothermophilus* strain *DSM 22* were purchased at the German Collection of Microorganisms and Cell Cultures (DSMZ) and grown in the corresponding recommended liquid medium. Cultivation was carried out at various growth temperatures in a culture shaker at 180 rpm and a volume of 50–100 mL. Cell growth was photometrically monitored and cells were harvested by centrifugation for 10 min at 4 °C and 3500× *g* once they had reached the mean log phase. Dependent on the organism and growth temperature, the OD600 was between 0.31 and 0.84.

### 4.2. Isolation of Total RNA and Small RNA Enrichment

Bacterial cell pellets were resuspended in TRIzol^TM^. Cell disruption was performed using a FastPrep homogenizer (MP Biomedicals, Eschwege, Germany) for 40 s at 6 m/s, followed by incubation at room temperature for 5 min. A total of 200 μL chloroform was added, mixed for 15 s, and incubated for 3 min at room temperature. After centrifugation for 15 min at 12,000× *g* and 4 °C, the supernatant was transferred to a sterile reaction tube, mixed with an identical volume of isopropanol, and incubated on ice for 15 min. RNA was pelleted by centrifugation and washed twice with 1 mL ethanol (70%). After air drying at 30 °C, the total RNA was dissolved in 200 μL sterile water. For small RNA enrichment, the total RNA preparation was mixed with 1/10 volume of 5 M NaCl and 50% PEG 8000 and incubated for 30 min at −20 °C. After centrifugation for 30 min at 10,000× *g* and 4 °C, the procedure was repeated with the supernatant. The resulting solution was mixed with 3 volumes of ethanol and incubated overnight at −20 °C. Small RNAs were precipitated by centrifugation and resuspended in water.

### 4.3. NaBH_4_-Treatment for the Detection of Dihydrouridine and 7-Methylguanosine

Treatment of the small RNA fraction was conducted as described in [63]. Amounts of 2–4 µg small RNA were adjusted with water to a volume of 50 µL, and 5 µL of a freshly prepared NaBH_4_ solution (100 mg/mL in 0.01 N KOH) was added. The mixture was incubated on ice for 30 min. The reaction was stopped by the addition of 5 µL 0.01 N acetic acid, followed by ethanol precipitation. The RNA was resuspended in sterile water and kept at −20 °C until use.

### 4.4. CMCT-Treatment for the Detection of Pseudouridine

The procedure was based on the protocol described in [14], with slight modifications. Briefly, 2–4 μg small RNA was mixed with 30 μL of freshly prepared 1-cyclohexyl-(2-morpholinoethyl)carbodiimide metho-p-toluene sulfonate (CMCT) solution (165 mM CMCT, 50 mM Bicine, pH 8.0, 7 M urea, 4mM EDTA) or CMCT buffer (solution without CMCT) as control and incubated for 20 min at 37 °C. The reaction was stopped by ethanol precipitation in the presence of 1 µg glycogen. The RNA pellet was resuspended in 40 µL 50 mM Na_2_CO_3_ solution (pH 10.3) and incubated for 2 h at 37 °C. After another ethanol precipitation, the pellet was dissolved in sterile water.

### 4.5. Adenylation of Adapter Oligonucleotide and Ligation to Small RNA Samples

A total of 500 pmol of a 3′-terminally blocked adapter oligonucleotide (5′-pTGGAATTCTCGGGTGCCAAGG-amino-C7-3′) was mixed with 50 pmol of recombinantly expressed and purified TS2126 RNA ligase (CircLigase) in 20 μL adenylation buffer (50 mM MOPS, pH 7.5, 10 mM KCl, 5 mM MgCl_2_) containing 2.5 mM MnCl_2_, 1 mM DTT, and 50 µM ATP and incubated for 2 h at 60 °C as previously described [64,65]. The reaction was stopped by heat inactivation at 80 °C for 10 min. Adenylated adapter was purified via phenol/chloroform extraction and ethanol precipitation and resuspended in water at a concentration of 20 µM. For ligation to the 3′-end of the prepared small RNA, 20 pmol of the adenylated adapter was incubated with 10 pmol sRNA sample in 10 µL 50 mM Tris/HCl, pH 7.5, 2 mM MgCl_2_, 1 mM DTT, 0.4 mM ATP, 20% PEG 8000, and 14 pmol recombinantly prepared T4 RNA ligase 2, truncated KQ (T4Rnl2KQ). After incubation at 25 °C for 20 h, the reaction was stopped by heat inactivation at 65 °C for 20 min.

### 4.6. Reverse Transcription

A total of 10 pmol phosphorylated RT primer (spiked with 1 pmol of 5′-^32^P-labeled RT primer for monitoring) was added to the adapter-ligated small RNA sample. Reverse transcription was performed with SuperScript IV according to the manufacturer’s instructions (Thermo Fisher Scientific, Waltham, MA, USA). The reaction was stopped by the addition of 1 μL 5 N NaOH and incubation at 95 °C for 3 min. The solution was neutralized by adding 1 μL 5 N HCl. A total of 8 μL 3× RNA loading buffer was added to the mixtures. The reaction products were separated on a 15% denaturing polyacrylamide gel, visualized by autoradiography, cut out using a sterile blade, and isolated according to Roth et al. [66]. As the RT primer (Supplemental Table S1) was used for circularization of the complementary DNA (cDNA), it contained two 18-atom hexa-ethylene glycol spacers (Sp18) to avoid rolling circle amplification during PCR [67,68,69]. In addition, the primer contained a binding site for the Illumina small RNA library primer for preparation of the sequencing library.

### 4.7. Circularization, Amplification and Indexing of cDNA

Gel-purified cDNA was incubated at 60 °C for 2 h in the presence of 17 pmol TS2126 RNA ligase (CircLigase) in a volume of 20 µL adenylation buffer supplemented with 5 mM MnCl_2_, 20 mM DTT, and 0.1 mM ATP. The ligation reaction was stopped by heat inactivation at 80 °C for 10 min. The individual sequencing libraries were prepared by PCR according to the Illumina TruSeq smallRNA library instructions. A total of 5 μL of circularized cDNA was added to a reaction volume of 50 μL containing 1× Phusion HF buffer, 0.2 mM dNTPs, 0.5 μM primer containing Illumina P5 sequence, 0.5 μM index primer, and 1 U of Phusion DNA polymerase (Thermo Fisher Scientific, USA). Each reaction was split and amplified in 10 to 12 cycles. PCR products were purified using the QIAquick PCR Purification Kit (Qiagen, Venlo, The Netherlands) according to the manufacturer’s instructions, and the concentration was determined photometrically. The P5-containing primer and primer containing the Illumina P7 sequence are listed in Supplementary Table S1.

### 4.8. High Throughput tRNA Sequencing

Single-end RNA sequencing (RNA seq; 1 × 100 bp) of the cDNA libraries was performed on an Illumina MiSeq device (Max Planck Institute for Evolutionary Anthropology, Leipzig, Germany) using the MiSeq Reagent Kit v3. Three biological replicates were sequenced for the NaBH_4_- and CMCT-treatment as well as for the corresponding untreated negative control for each bacterium and cultured temperature (in total 36 RNA seq libraries per species).

### 4.9. Annotation of tRNAs

Cytosolic tRNAs were annotated with *tRNAscan-SE* v2.0 [70] using the default model for bacteria. Obtained tRNA sequences were aligned to the tRNAdb [38] database using *BLAST* v2.4.0 [71] to fit individual tRNA secondary structure annotation to the corresponding standard tRNA model [72].

### 4.10. Mapping of tRNA Reads

To trim adapter sequences and low-quality portions of raw reads, Cutadapt v1.16 [73] was applied with a quality cutoff of 25 and a maximum error rate of 0.15. Only reads of 8 to 95 nts length (after trimming of adapter and low-quality bases) were captured. Read mapping and filtering were performed following the best-practice tRNA mapping strategy as described [74]. In brief, the reads were mapped against a modified target genome in which known tRNA loci were masked and instead intronless tRNA precursor sequences were appended as artificial “chromosomes”. In a first pass, reads displaying specific precursor hallmarks were filtered out. In the second pass, the mature tRNA reads were mapped against mature tRNA sequence clusters that assembled identical tRNAs, helping to overcome the multi-copy nature of tRNA genes. Consequently, only uniquely mapped high confidence tRNA reads were used for downstream analyses. The genomes of the four bacteria of the corresponding strain were obtained from the database of the National Center for Biotechnology Information (NCBI) [75].

### 4.11. Library Normalization

For direct comparisons of control and treated libraries, mapped data were scaled library- and replica-wise. Library-wise normalization was performed by scaling the number of mapped reads for each tRNA cluster position to the total sample number of reads. For replica-wise normalization, the mean value of replicates was calculated for each genomic tRNA position.

### 4.12. Identification of Significant Base Misincorporation Sites

For detection of tRNA modifications directly visible as accumulation of nucleotide mismatches in mapped reads, bcftools v1.15.1 [76] was used. First, the mpileup command of bcftools was applied to generate genotype probabilities and read coverage at each genomic position. In a second step, the variants were read out using the call command with the *-m* option to determine rare variants to be included. Variants below the Phred-scaled confidence threshold of 20 and with a coverage of less than 10 reads were excluded.

### 4.13. Profiling Position-Wise RT-Stops

To identify whether a particular position in a tRNA was significantly altered by chemical treatment, the difference in the number of normalized RT-stop sites in the treated versus untreated libraries was determined. Drawing on the framework used in differential gene expression analysis, a fold change (FC) was calculated at first to differentially quantify effect sizes in RT-stop coverage between the treatment and control samples and test for each tRNA position the null hypothesis that there is no significant change between both conditions. The FC for each tRNA and position *n* was calculated as

(1) $$FC_{n}=S{top}_{n+1}^{+}/\left(\gamma {Stop}_{n+1}^{-}+\alpha \right)$$

where *Stop^+^* and *Stop^−^* are the number of RT-stops of the treated and untreated libraries, respectively. In case of modified tRNAs, the RT mostly terminates one position upstream of the modified nucleotide. Thus, the values of ${Stop}_{n+1}^{+}$ and ${Stop}_{n+1}^{-}$ pertain to the chemical modification at position *n*, since the RNA template is read in the 3′ to 5′ direction during RT. For between-sample normalization, the untreated library was scaled by the ratio γ of total number of reads mapped per tRNAs cluster as a scaling factor given by

(2) $$\gamma =\Sigma Reads_{clust}^{+}\;/\;\Sigma \;Reads_{clust}^{-}.$$

To handle complications arising from zero counts, a pseudocount of α = 1 was added.

We do not expect or observe substantial overdispersion and hence model the sampling of read ends (caused by RT-stops) as a Poisson process [77] as second step. Since we expect an enrichment of RT-stops only in the treated samples, the RT-stops derived from the untreated libraries were used to estimate the expected background signal as $\lambda =\gamma \backslash RT_{n+1}^{-}$. Since the expression values are different for individual tRNAs (more precisely for the clusters of tRNAs with nearly Indistinguishable sequences), λ is estimated separately for each tRNA cluster. This additionally reduced the influence of potential outliers. Subsequently, the *p* value for the observation of RT-stops *k* (including the pseudo number) was calculated for each tRNA position as follows:

(3) $$P\left(x\geq k\right)=P_{\lambda }\left(k\right)=1-\left(\frac{\lambda^{k}}{k!}e^{-\lambda }\right)\;$$

To account for multiple testing, the false discovery rate (FDR) was calculated by applying the Benjamini–Hochberg procedure [78]. Only tRNA positions with an FDR adjusted *p* value of less than 0.01 were considered as significantly modified.

### 4.14. Choice of Cutoff Values to Reduce Noise

However, regardless of filtering, a large amount of presumably spurious noise remains. To further reduce the latter, three different parameters were considered:

1. *Fold change (FC)*: A sufficiently large effect size (FC) is required, i.e., position-specific increase of the *chemically* introduced RT-stops in treated compared to the untreated library.
2. *Total number of RT-stops*: To reduce signals with an overestimated log_2_ FC, which can arise at positions with a low read coverage even at a few RT-stops.

*Percentage of RT-stops*: Treatment-based RT-stops have a higher ratio of reads with RT-stops to reads without RT-stops at the corresponding position compared to noise.

Therefore, an appropriate percentage of RT-stops helps to reduce noise. The thresholds were set as follows: FC > 1; total number of RT-stops ≥ 20; and percentage of RT-stops ≥ 2.

### 4.15. Outcome Comparisons with mim-tRNAseq

For the comparison study, *mim-tRNAseq* v1.1.6 [41] was utilized. A *misinc-thresh* threshold (total misincorporation rate at a position in a cluster used to call unannotated modification) of 0.005 was used to reduce noise. All other parameters were set according to default settings.

### 4.16. MS/MS Analyses

#### 4.16.1. tRNA Cyanoethylation

tRNAs were chemically treated with acrylonitrile to target 4-thiouridine (s^4^U) modifications. The acrylonitrile derivatizations (Ces^4^U for s^4^U) result in a nucleotide mass increase of +53.0 Da, which can be readily detected using mass spectrometry (MS) [79,80].

#### 4.16.2. tRNA Isolation by 2D-Gel Electrophoresis

tRNA isoacceptors were isolated using two-dimensional gel electrophoresis as described in [81]. In short, tRNA was briefly heated to 90 °C. Then, samples were separated in a first-dimension 12.5% polyacrylamide gel containing 1× TBE and 8 M urea under denaturing conditions, followed by a second dimension under semi-denaturing conditions using a 20% polyacrylamide gel, TBE 1×, and 4 M urea at room temperature. Gels were stained with an ethidium bromide solution (10 µg/L) for 10 min. Spots containing isolated tRNA isoacceptors were visualized and excised under UV light (302 nm).

#### 4.16.3. In-Gel RNase Digestion of tRNAs

Gel spots containing tRNAs were desalted by at least eight washes with 100 µL 200 mM NH_4_AcO and dried under vacuum. For RNase T_1_ hydrolysis, gel pieces were rehydrated by 20 µL of 0.1 U/µL of RNase T_1_ or by 20 µL of 0.01 U/µL of RNase A (ThermoFisher Scientific) in 100 mM NH_4_AcO (pH not adjusted). For RNase U_2_ treatment, spots were digested by using 50 µL of recombinant RNase U_2_ at 0.3 ng/µL in 50 mM NH_4_AcO (pH 5.3) and incubated for 45 min at 65 °C. After digestion, supernatants were dried under vacuum.

#### 4.16.4. Nano Liquid Chromatography-MS/MS of RNA Oligonucleotides and Analysis

Pellets containing RNase digestion products were resuspended in 3 µL of milli-Q water and separated on an Acquity peptide BEH C18 column (130 Å, 1.7 µm, 75 µm × 200 mm) using a nanoAcquity system (Waters, Milford, MA, USA). The analyses were performed with an injection volume of 3 µL. The column was equilibrated in eluant A containing 7.5 mM TEAA (Triethylammonium acetate), 7.0 mM TEA (Triethylammonium), and 200 mM HFIP (hexafluoroisopropanol) at a flow rate of 300 nL/min. Oligonucleotides were eluted using a gradient from 15% to 35% of eluant B (100% LC/MS Grade methanol) for 2 min followed by an increase of buffer B to 50% in 20 min. MS and tandem mass spectrometry (MS/MS) analyses were performed using SYNAPT G2-S (hybrid quadrupole time-of-flight mass spectrometer) from Waters Corporation. All experiments were performed in negative mode with a capillary voltage set at 2.6 kV and a sample cone voltage set at 30 V. The source was heated to 130 °C. The samples were analyzed over an *m*/*z* range from 550 to 1600 for the full scan, followed by a fast data direct acquisition scan (Fast DDA). Collision-induced dissociation (CID) experiments were achieved using Ar. All MS/MS spectra were deconvoluted using MassLynx software v4.1 (Waters) and manually sequenced by following the y and/or c series (w ions were also used when sequencing was difficult or to confirm a sequence). tRNA identification was performed via comparisons with the genomic sequences.

## 5. Conclusions

In conclusion, this study provides insights into both short-term temperature-dependent changes and long-term evolutionary adaptations in tRNA modification patterns within bacterial organisms through specific modifications at a single-nucleotide resolution. Our established computational pipeline for identifying chemical modifications in RNA sequencing data proved highly effective, requiring minimal parameters and showing promise for accurate profiling studies. Importantly, we were able to construct a comprehensive tRNA modification profile for the four bacteria, particularly enhancing our understanding of *E. sibiricum* and *P. halocryophilus*, where limited information was previously available. Furthermore, we successfully identified Ψ at position U60 in the four bacteria through both our MS/MS and RNA seq analyses, revealing the presence of Ψ at this position in bacteria where it was previously unknown. Our results indicate that the thermophilic *G. stearothermophilus* displayed a clear temperature-dependent increase in specific tRNA modifications (D17, D20, D20a, Ψ39, and Ψ55) at higher temperatures, suggesting a regulatory mechanism linked to temperature variations. The elevated presence of s^4^U8 and Ψ55 modifications in *G. stearothermophilus* compared to non-thermophilic bacteria may act as adaptive strategies for maintaining tRNA stability and functionality under extreme high-temperature conditions. Moreover, the higher abundance of D modifications in tRNAs from psychrophilic and mesophilic bacteria implies an essential adaptation to cold environments by enhancing local flexibility in tRNAs.

## Figures and Tables

**Table 1:** Overview of tRNA modifications listed for different bacterial growth temperatures. tRNA cluster numbers of s^4^U, D, m^7^G, and Ψ tRNA modifications identified at specific tRNA positions across three measured temperatures in four bacterial strains.

	*P. halocryophilus*			*E. sibiricum*			*B. subtilis*			*G. stearothermophilus*		
	10 °C	20 °C	30 °C	10 °C	20 °C	30 °C	20 °C	30 °C	37 °C	40 °C	55 °C	70 °C
s^4^U8	1	3	2	3	-	1	4	4	4	30	32	24
D16	5	6	5	3	3	3	7	4	4	-	3	3
D17	14	14	15	15	16	16	20	21	21	1	12	13
D20	17	17	17	25	23	24	21	21	19	6	18	19
D20a	12	12	12	11	11	11	14	14	14	2	10	11
D20b	-	-	-	-	1	-	-	-	-	-	-	-
Ψ31	1	1	1	2	2	2	3	3	2	2	2	5
Ψ32	-	-	1	1	2	3	1	1	1	1	2	2
Ψ38	2	2	2	1	1	1	-	-	-	-	-	-
Ψ39	11	11	11	13	13	14	13	13	13	9	13	14
m^7^G46	17	17	17	18	18	18	22	22	23	18	18	18
D47	-	3	1	14	4	7	-	-	10	2	2	5
Ψ55	24	20	21	20	31	31	27	29	25	9	22	29
Ψ60	-	5	13	-	-	4	-	12	-	-	12	10

## Data Availability

All data from this study are contained within the published article and its supplementary information files. The RNA seq data can be accessed at NCBI’s Sequence Read Archive [82] under BioProject ID PRJNA1077126 (https://www.ncbi.nlm.nih.gov/bioproject/PRJNA1077126, accessed on 10 July 2024).

## Supplementary Materials

The following supporting information can be downloaded at: https://www.mdpi.com/article/10.3390/ijms25168823/s1.
