# Supplementary material for: Temperature-Dependent tRNA Modifications in Bacillales
Source: Int J Mol Sci. 2024 Aug 13;25(16):8823. doi: 10.3390/ijms25168823 (PMC11354880; doi:10.3390/ijms25168823)
Supplement: Supplementary file 1 [file ijms-25-08823-s001.zip › Supplementary Figures S1-S12.pdf]

SUPPLEMENTARY MATERIALS

Temperature-dependent tRNA Modifications in Bacillales

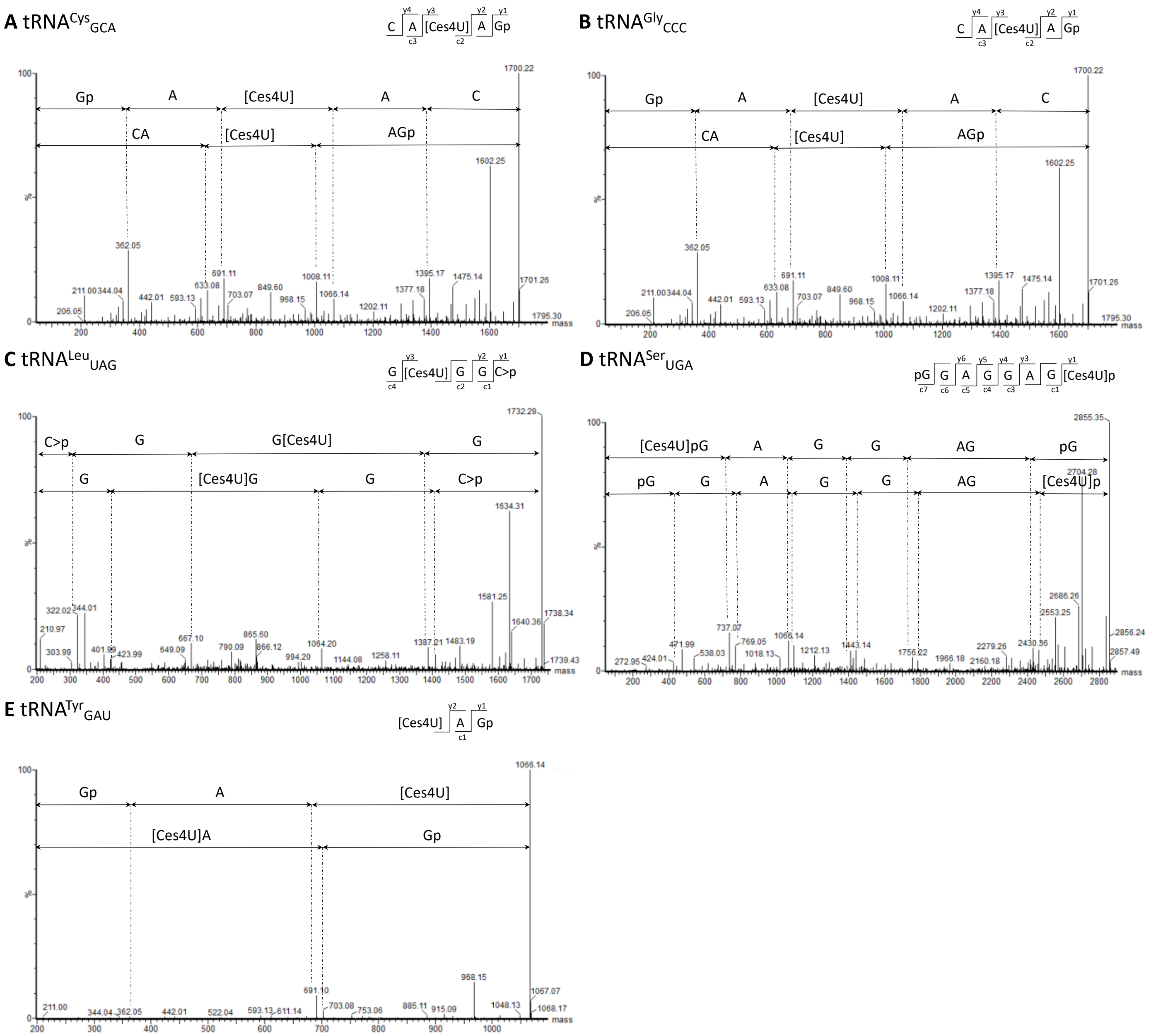

Supplementary Figure S1. Additional MS/MS sequencing spectra containing cyanoethylated 4-thiouridine (Ces^{4}U) at tRNA position U8 of *G. stearothermophilus*. A) MS/MS spectrum CA[Ces^{4}U]AGp of tRNA$^{Cys}_{GCA}$ after RNase T$_1$ digestion (*m/z* 849.61 z = 2-). **B)** MS/MS spectrum UG[Ces^{4}U]A>p of tRNA$^{Gly}_{CCC}$ after RNase U$_2$ digestion (*m/z* 676,56 z = 2-). **C)** MS/MS spectrum G[Ces^{4}U]GGC>p of tRNA$^{Leu}_{UAG}$ after RNase A digestion (*m/z* 865.63 z = 2-). **D)** MS/MS spectrum pGGAGGAG[Ces^{4}U]p of tRNA$^{Ser}_{UGA}$ after RNase A digestion (*m/z* 1427.18 z = 2-). **E)** MS/MS spectrum [Ces^{4}U]AGp of tRNA$^{Tyr}_{GAU}$ after RNase T$_1$ digestion (*m/z* 1066.13 z = 1-).

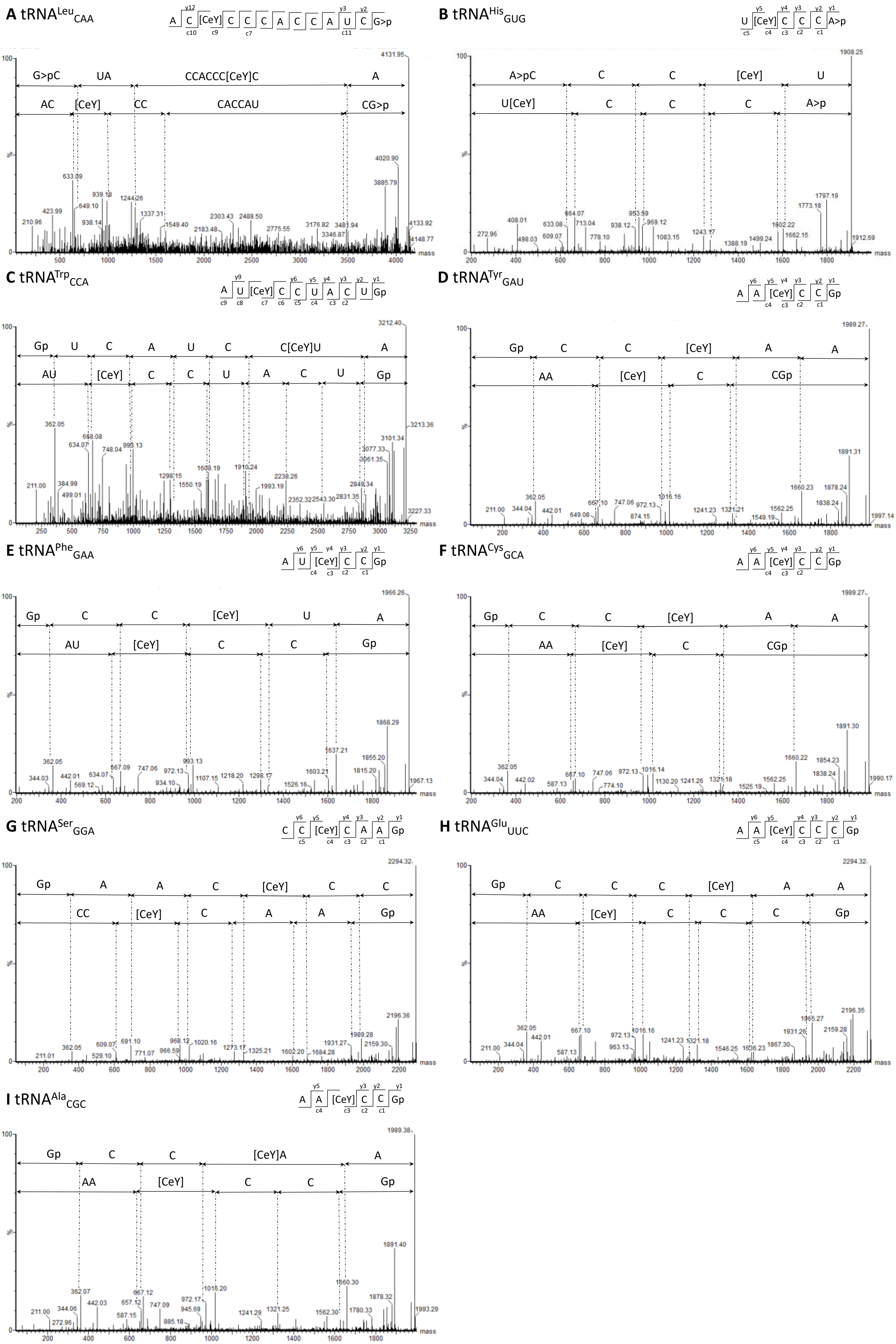

A tRNA$^{Leu}_{CAA}$
A C [CeY] C C A C C A U C G>p
G>pC UA CCACCC[CeY]C A
AC [CeY] CC CACCAU CG>p
B tRNA$^{His}_{GUG}$
U [CeY] C C C A>p
A>pC C C [CeY] U
U[CeY] C C C A>p
C tRNA$^{Trp}_{CCA}$
A U [CeY] C C U A C U Gp
Gp U C A U C C[CeY]U A
AU [CeY] C C U A C U Gp
D tRNA$^{Tyr}_{GAU}$
A A [CeY] C C Gp
Gp C C [CeY] A A
AA [CeY] C CGp
E tRNA$^{Phe}_{GAA}$
A U [CeY] C C Gp
Gp C C [CeY] U A
AU [CeY] C C Gp
F tRNA$^{Cys}_{GCA}$
A A [CeY] C C Gp
Gp C C [CeY] A A
AA [CeY] C CGp
G tRNA$^{Ser}_{GGA}$
C C [CeY] C A A Gp
Gp A A C [CeY] C C
CC [CeY] C A A Gp
H tRNA$^{Glu}_{UUC}$
A A [CeY] C C C Gp
Gp C C C [CeY] A A
AA [CeY] C C C Gp
I tRNA$^{Ala}_{CGC}$
A A [CeY] C C Gp
Gp C C [CeY]A A
AA [CeY] C C Gp
mass

Supplementary Figure S2. MS/MS sequencing spectra containing cyanoethylated pseudouridine (CeΨ) at tRNA position U60 of *G. stearothermophilus*. A) MS/MS spectrum AC[CeΨ]CCCACCAUCGp of $tRNA^{Leu}_{CAA}$ after RNase T_1 digestion (*m/z* 1376.64 z = 2-). **B)** MS/MS spectrum U[CeΨ]CCCA>p of $tRNA^{His}_{GUG}$ after RNase U_2 digestion (*m/z* 953.62 z = 2-). **C)** MS/MS spectrum AU[CeΨ]CCUACUGp of $tRNA^{Trp}_{CCA}$ after RNase T_1 digestion (*m/z* 1070.13 z = 2-). **D)** MS/MS spectrum AA[CeΨ]CCGp of $tRNA^{Tyr}_{GAU}$ after RNase T_1 digestion (*m/z* 994.14 z = 2-). **E)** MS/MS spectrum AU[CeΨ]CCGp of $tRNA^{Phe}_{GAA}$ after RNase T_1 digestion (*m/z* 982.62 z = 2-). **F)** MS/MS spectrum AA[CeΨ]CCGp of $tRNA^{Cys}_{GCA}$ after RNase T_1 digestion (*m/z* 994.14 z = 2-). **G)** MS/MS spectrum CC[CeΨ]CAAGp of $tRNA^{Ser}_{GGA}$ after RNase T_1 digestion (*m/z* 1146.65 z = 2-). **H)** MS/MS spectrum AA[CeΨ]CCCGp of $tRNA^{Glu}_{UUC}$ after RNase T_1 digestion (*m/z* 1146.67 z = 2-). **I)** MS/MS spectrum AA[CeΨ]CCGp of $tRNA^{Ala}_{CGC}$ after RNase T_1 digestion (*m/z* 994.19 z = 2-).

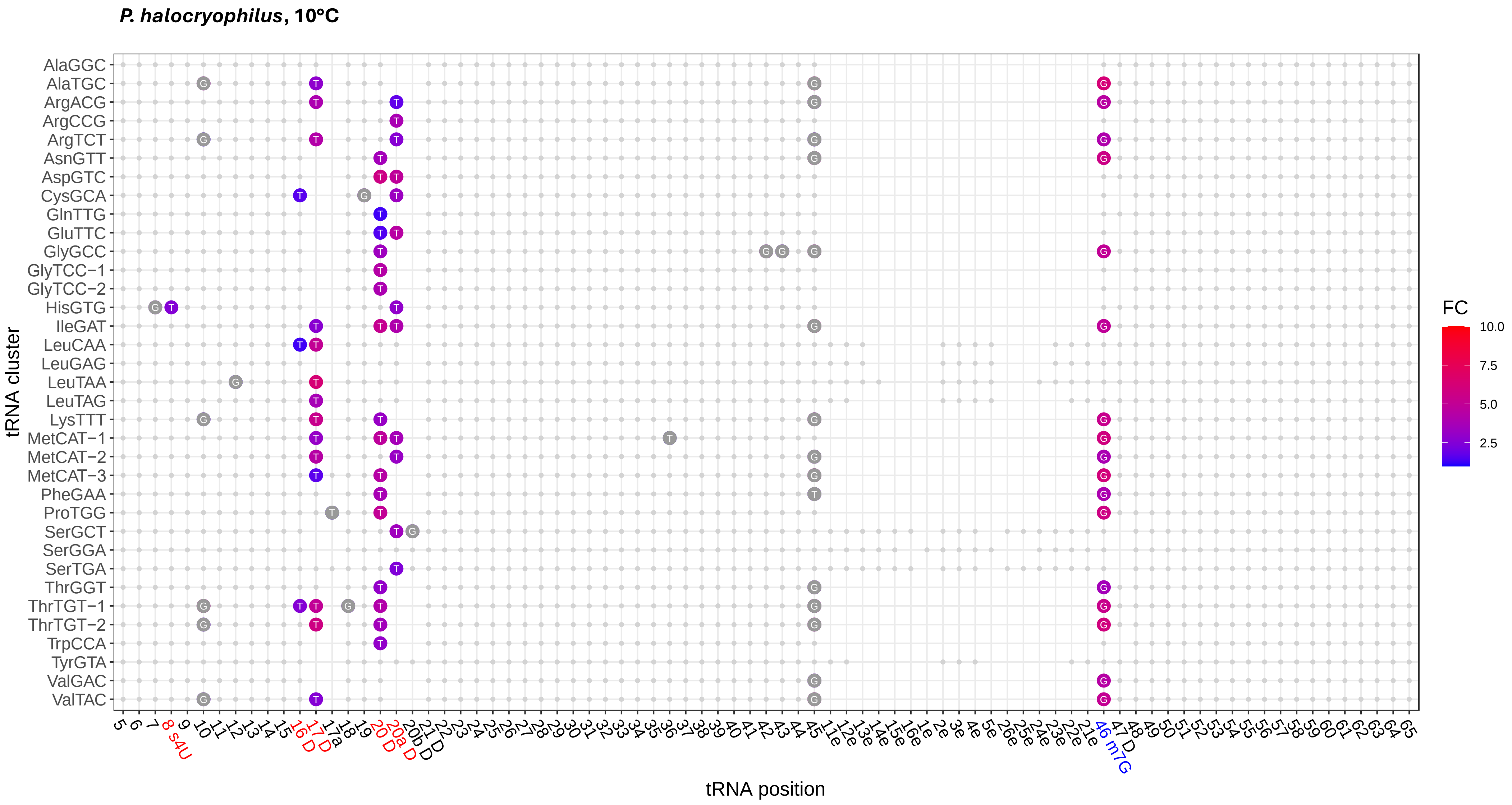

P. halocryophilus, 10°C
tRNA cluster
tRNA position
FC
10.0
7.5
5.0
2.5
AlaGGC
AlaTGC
ArgACG
ArgCCG
ArgTCT
AsnGTT
AspGTC
CysGCA
GlnTTG
GluTTC
GlyGCC
GlyTCC−1
GlyTCC−2
HisGTG
IleGAT
LeuCAA
LeuGAG
LeuTAA
LeuTAG
LysTTT
MetCAT−1
MetCAT−2
MetCAT−3
PheGAA
ProTGG
SerGCT
SerGGA
SerTGA
ThrGGT
ThrTGT−1
ThrTGT−2
TrpCCA
TyrGTA
ValGAC
ValTAC
8 s4U
16 D
17 D
20 D
20a D
46 m7G

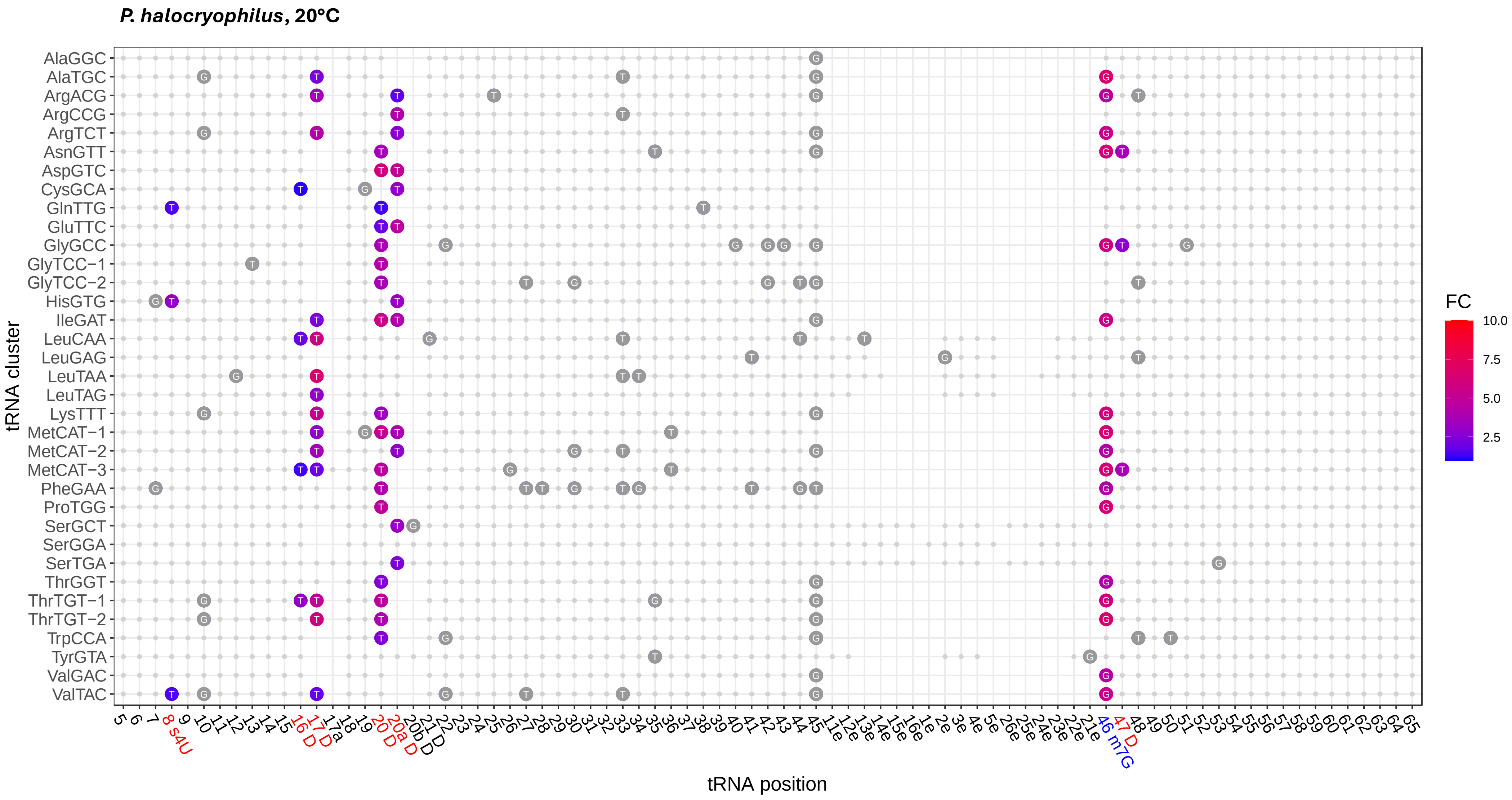

P. halocryophilus, 20°C
tRNA cluster
tRNA position
FC
10.0
7.5
5.0
2.5
AlaGGC
AlaTGC
ArgACG
ArgCCG
ArgTCT
AsnGTT
AspGTC
CysGCA
GlnTTG
GluTTC
GlyGCC
GlyTCC−1
GlyTCC−2
HisGTG
IleGAT
LeuCAA
LeuGAG
LeuTAA
LeuTAG
LysTTT
MetCAT−1
MetCAT−2
MetCAT−3
PheGAA
ProTGG
SerGCT
SerGGA
SerTGA
ThrGGT
ThrTGT−1
ThrTGT−2
TrpCCA
TyrGTA
ValGAC
ValTAC
8 s4U
16 D
17 D
20 D
20a D
20b D
46 m7G
47 D

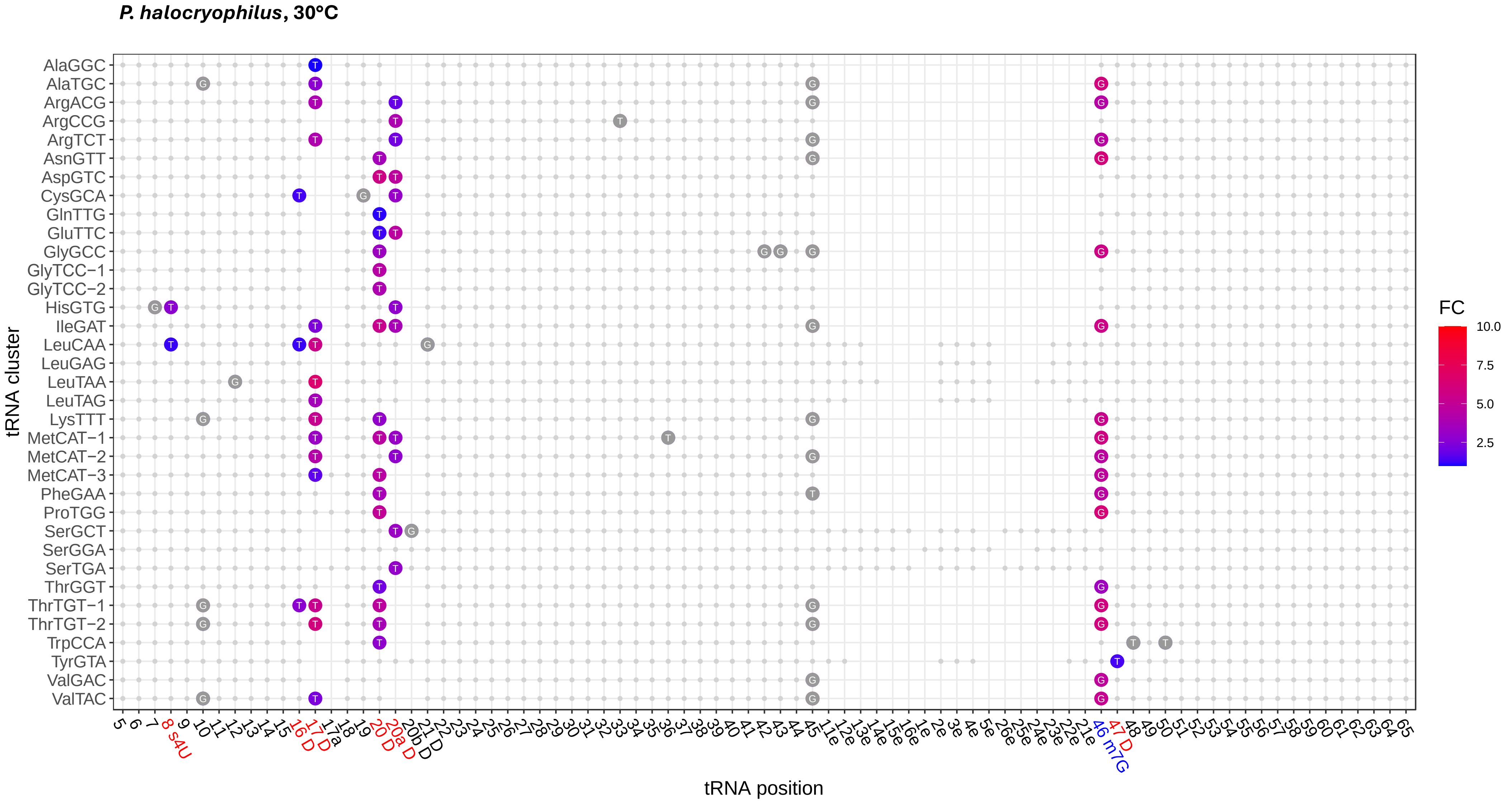

Supplementary Figure S3. Read termination sites from $NaBH_4$-treated RNA seq data of *P. halocryophilus*. The figure illustrates the investigated read termination (RT) sites for each tRNA cluster and tRNA position of *P. halocryophilus* at each growth temperature studied. All tRNA positions exhibiting a significant (adj. P value < 0.01) and strong (fold change (FC) ≥ 1, total number of RTs ≥ 20, and percentage of RTs ≥ 2) RT sites are color-coded from blue to red based on the logarithmic FC if the RT sites are classified as true positives. Type I false positive points are colored in gray. tRNA sites with no RT enrichment are represented as smaller gray dots. Enhanced RT sites were identified by comparing the RNA seq mapping profiles of sodium borohydride ($NaBH_4$)-treated samples with those of untreated control samples.

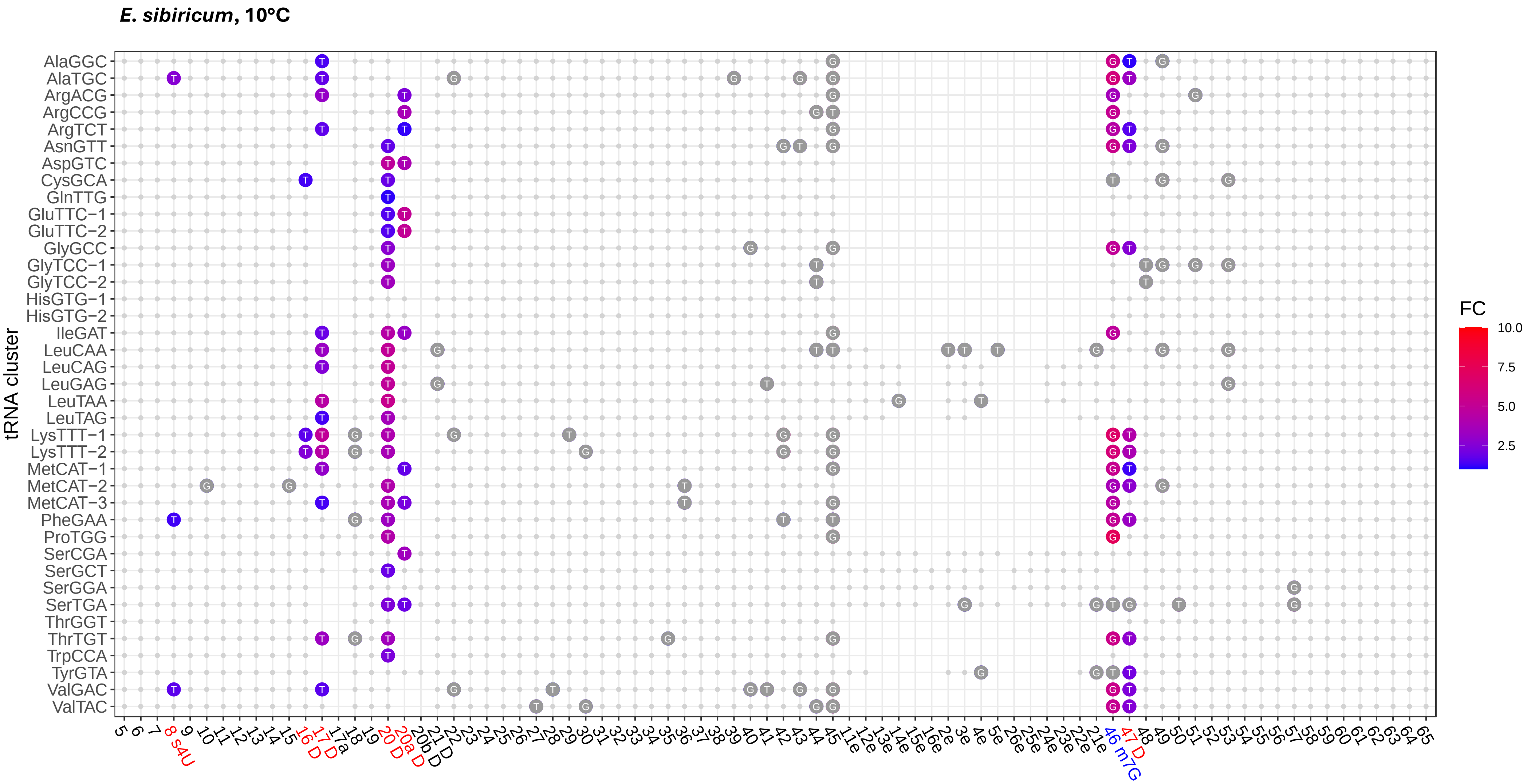
E. sibiricum, 10°C
tRNA cluster
AlaGGC
AlaTGC
ArgACG
ArgCCG
ArgTCT
AsnGTT
AspGTC
CysGCA
GlnTTG
GluTTC−1
GluTTC−2
GlyGCC
GlyTCC−1
GlyTCC−2
HisGTG−1
HisGTG−2
IleGAT
LeuCAA
LeuCAG
LeuGAG
LeuTAA
LeuTAG
LysTTT−1
LysTTT−2
MetCAT−1
MetCAT−2
MetCAT−3
PheGAA
ProTGG
SerCGA
SerGCT
SerGGA
SerTGA
ThrGGT
ThrTGT
TrpCCA
TyrGTA
ValGAC
ValTAC
5 6 7 8 s4U 9 10 11 12 13 14 15 16 D 17 D 17a 18 19 20 D 20a D 20b D 21 22 23 24 25 26 27 28 29 30 31 32 33 34 35 36 37 38 39 40 41 42 43 44 45 11e 12e 13e 14e 15e 16e 2e 3e 4e 5e 26e 25e 24e 23e 22e 21e 46 m7G 47 D 48 49 50 51 52 53 54 55 56 57 58 59 60 61 62 63 64 65
tRNA position
FC
10.0
7.5
5.0
2.5

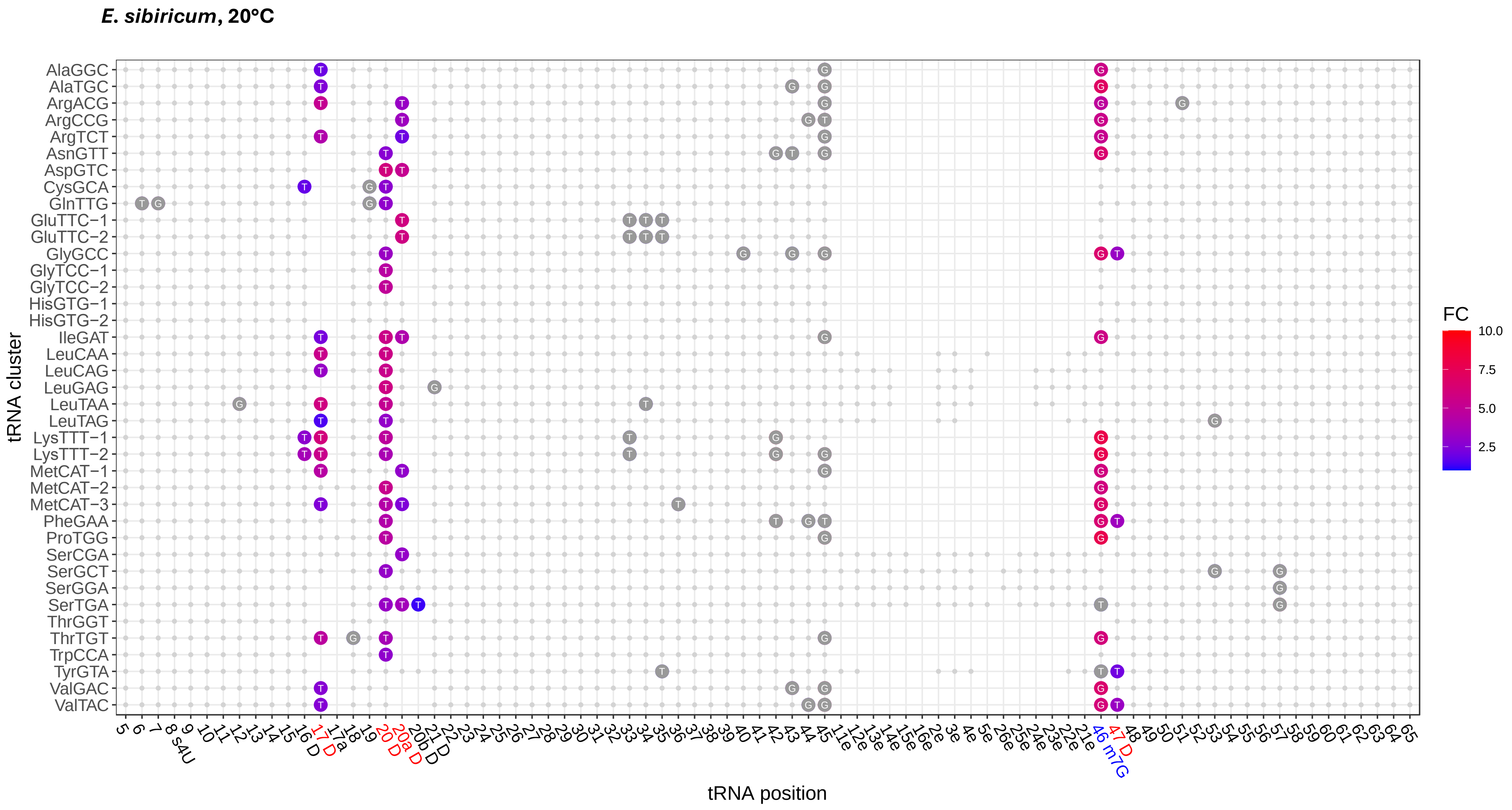

E. sibiricum, 20°C
tRNA cluster
AlaGGC
AlaTGC
ArgACG
ArgCCG
ArgTCT
AsnGTT
AspGTC
CysGCA
GlnTTG
GluTTC−1
GluTTC−2
GlyGCC
GlyTCC−1
GlyTCC−2
HisGTG−1
HisGTG−2
IleGAT
LeuCAA
LeuCAG
LeuGAG
LeuTAA
LeuTAG
LysTTT−1
LysTTT−2
MetCAT−1
MetCAT−2
MetCAT−3
PheGAA
ProTGG
SerCGA
SerGCT
SerGGA
SerTGA
ThrGGT
ThrTGT
TrpCCA
TyrGTA
ValGAC
ValTAC
tRNA position
8 s4U
16 D
17 D
20 D
20a D
20b D
46 m7G
47 D
FC
10.0
7.5
5.0
2.5

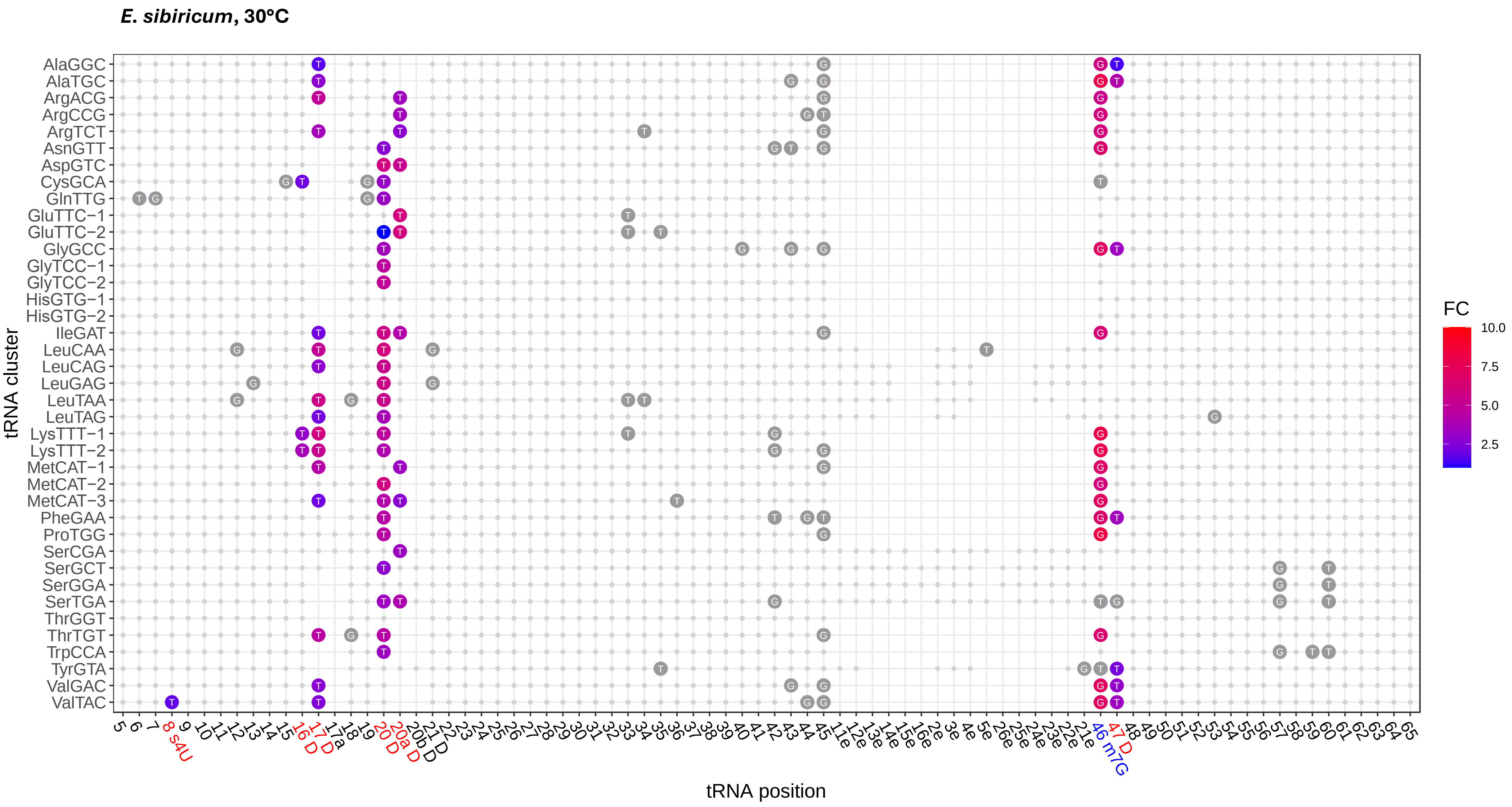

Supplementary Figure S4. Read termination sites from $NaBH_4$-treated RNA seq data of *E. sibiricum*. The figure illustrates the investigated RT sites for each tRNA cluster and tRNA position of *E. sibiricum* at each growth temperature studied. All tRNA positions exhibiting a significant (adj. P value < 0.01) and strong (FC ≥ 1, total number of RTs ≥ 20, and percentage of RTs ≥ 2) RT sites are color-coded from blue to red based on the logarithmic FC if the RT sites are classified as true positives. Type I false positive points are colored in gray. tRNA sites with no RT enrichment are represented as smaller gray dots. Enhanced RT sites were identified by comparing the RNA seq mapping profiles of $NaBH_4$-treated samples with those of untreated control samples.

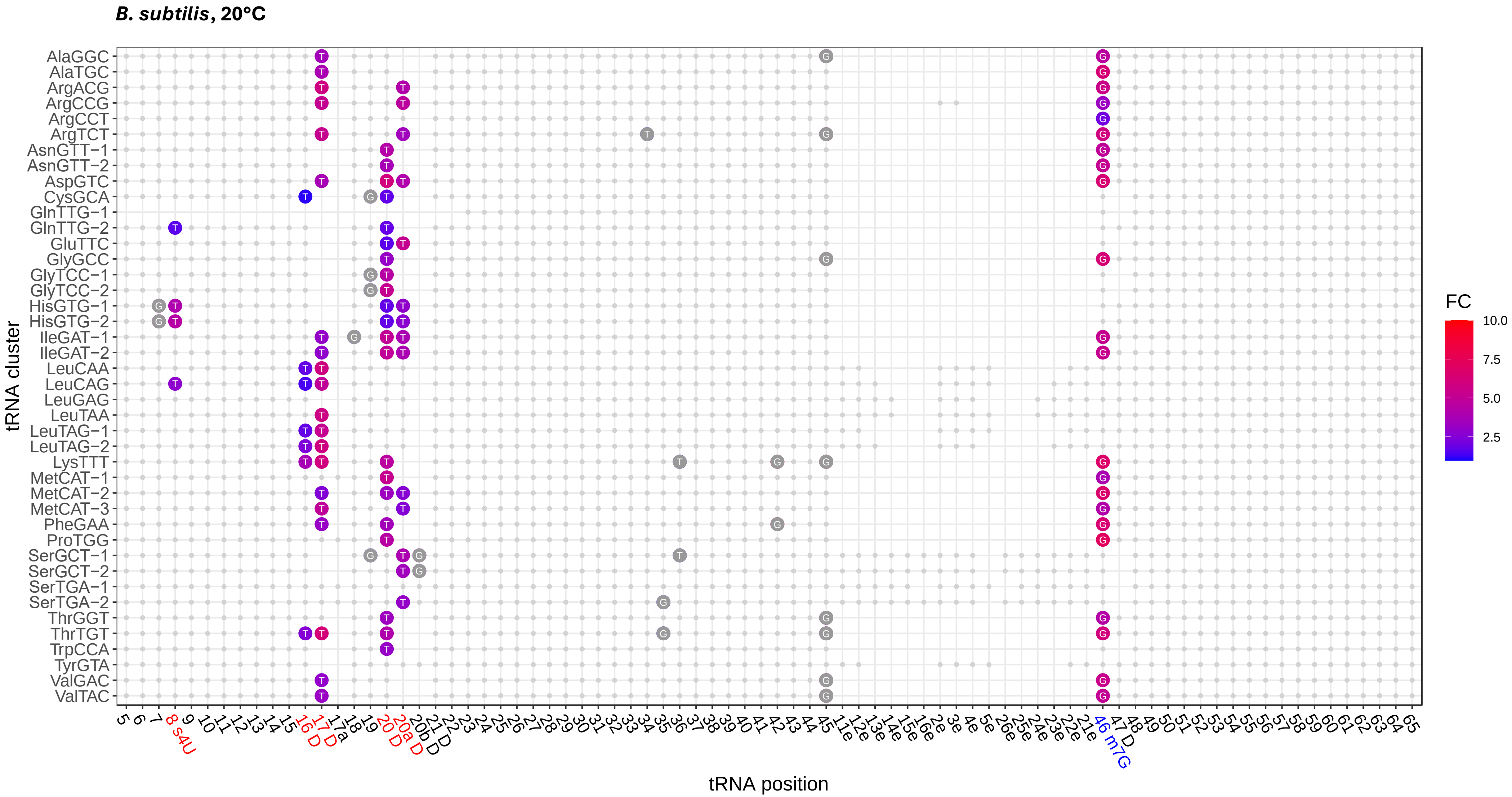
B. subtilis, 20°C
tRNA cluster
AlaGGC
AlaTGC
ArgACG
ArgCCG
ArgCCT
ArgTCT
AsnGTT−1
AsnGTT−2
AspGTC
CysGCA
GlnTTG−1
GlnTTG−2
GluTTC
GlyGCC
GlyTCC−1
GlyTCC−2
HisGTG−1
HisGTG−2
IleGAT−1
IleGAT−2
LeuCAA
LeuCAG
LeuGAG
LeuTAA
LeuTAG−1
LeuTAG−2
LysTTT
MetCAT−1
MetCAT−2
MetCAT−3
PheGAA
ProTGG
SerGCT−1
SerGCT−2
SerTGA−1
SerTGA−2
ThrGGT
ThrTGT
TrpCCA
TyrGTA
ValGAC
ValTAC
8 s4U
16 D
17 D
20 D
20a D
20b D
46 m7G
47 D
tRNA position
FC
10.0
7.5
5.0
2.5

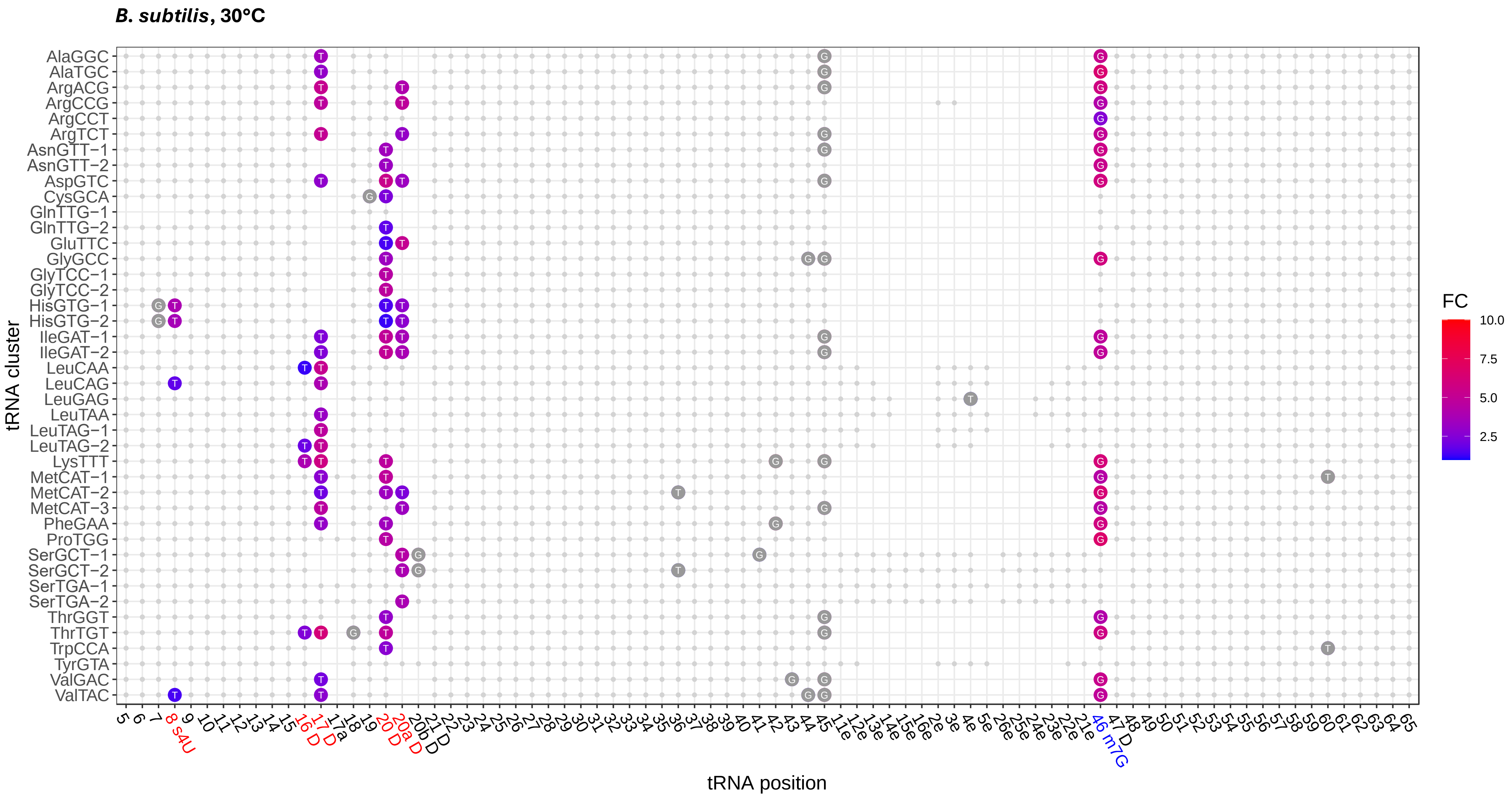

B. subtilis, 30°C
tRNA cluster
AlaGGC
AlaTGC
ArgACG
ArgCCG
ArgCCT
ArgTCT
AsnGTT−1
AsnGTT−2
AspGTC
CysGCA
GlnTTG−1
GlnTTG−2
GluTTC
GlyGCC
GlyTCC−1
GlyTCC−2
HisGTG−1
HisGTG−2
IleGAT−1
IleGAT−2
LeuCAA
LeuCAG
LeuGAG
LeuTAA
LeuTAG−1
LeuTAG−2
LysTTT
MetCAT−1
MetCAT−2
MetCAT−3
PheGAA
ProTGG
SerGCT−1
SerGCT−2
SerTGA−1
SerTGA−2
ThrGGT
ThrTGT
TrpCCA
TyrGTA
ValGAC
ValTAC
8 s4U
16 D
17 D
20 D
20a D
20b D
46 m7G
47 D
tRNA position
FC
10.0
7.5
5.0
2.5

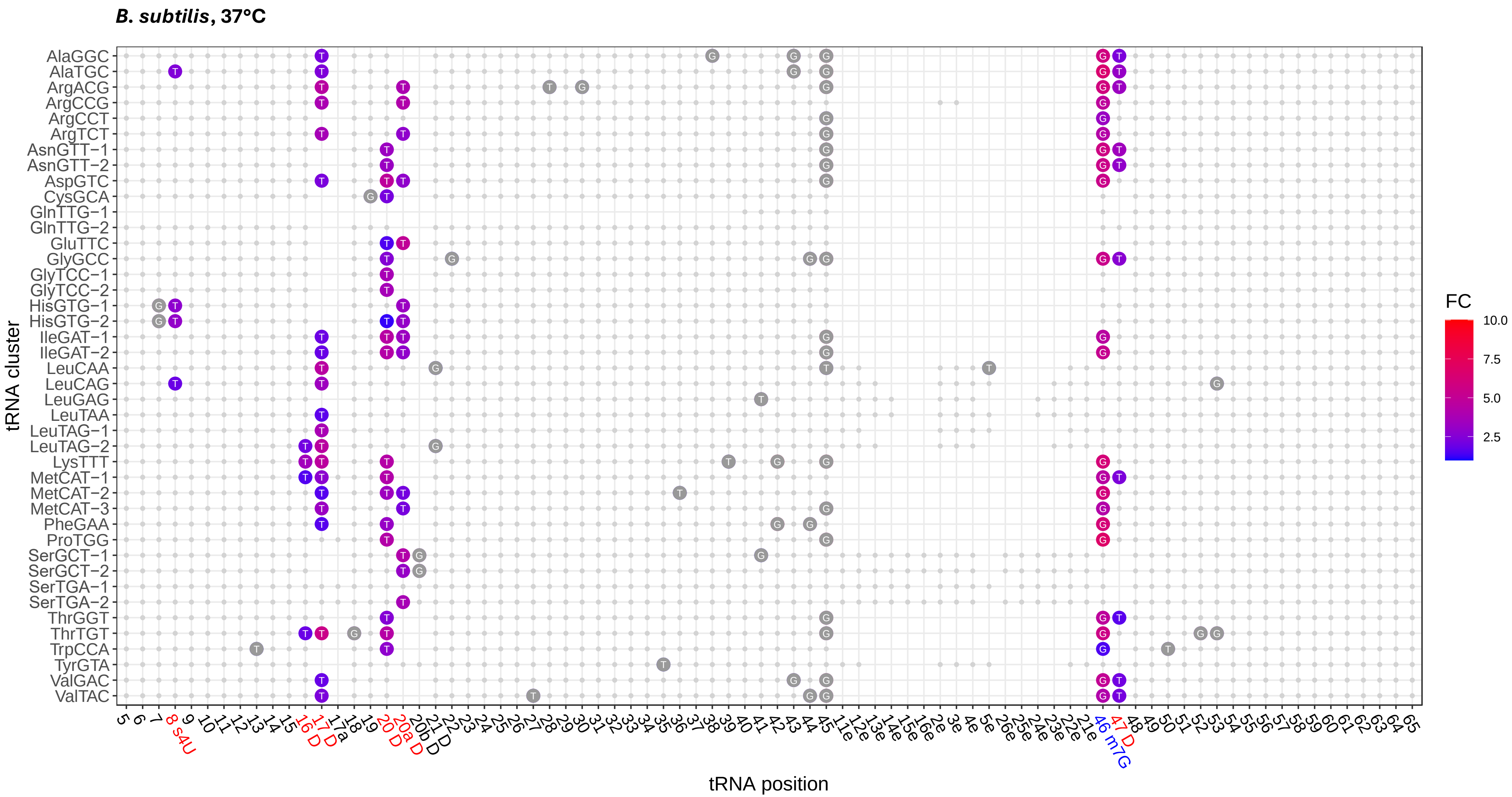

Supplementary Figure S5. Read termination sites from $NaBH_4$-treated RNA seq data of *B. subtilis*. The figure illustrates the investigated RT sites for each tRNA cluster and tRNA position of *B. subtilis* at each growth temperature studied. All tRNA positions exhibiting a significant (adj. P value < 0.01) and strong (FC ≥ 1, total number of RTs ≥ 20, and percentage of RTs ≥ 2) RT sites are color-coded from blue to red based on the logarithmic FC if the RT sites are classified as true positives. Type I false positive points are colored in gray. tRNA sites with no RT enrichment are represented as smaller gray dots. Enhanced RT sites were identified by comparing the RNA seq mapping profiles of $NaBH_4$-treated samples with those of untreated control samples.

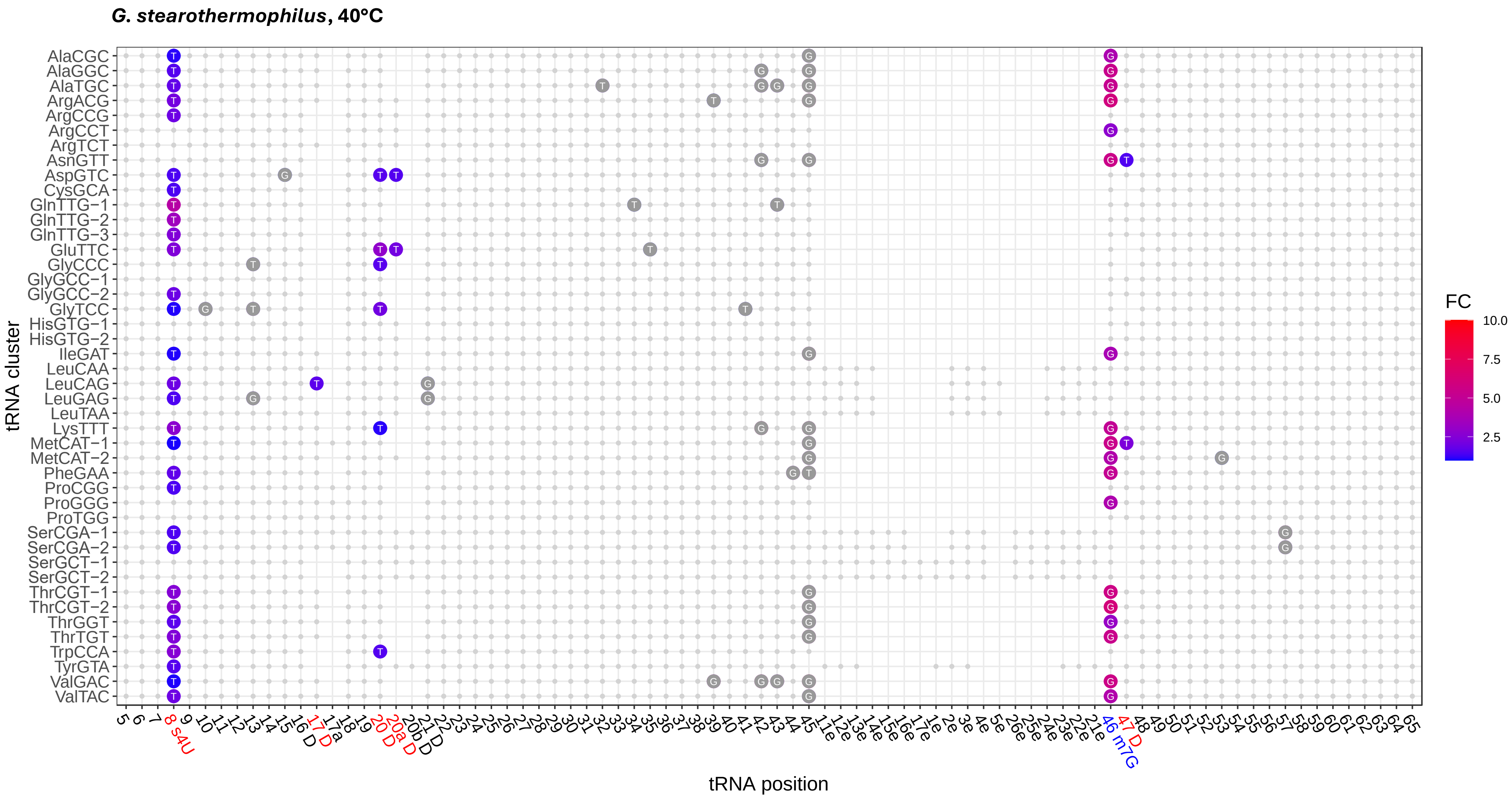

G. stearothermophilus, 40°C
tRNA cluster
tRNA position
FC
10.0
7.5
5.0
2.5
8 s4U
17 D
20 D
20a D
46 m7G
47 D

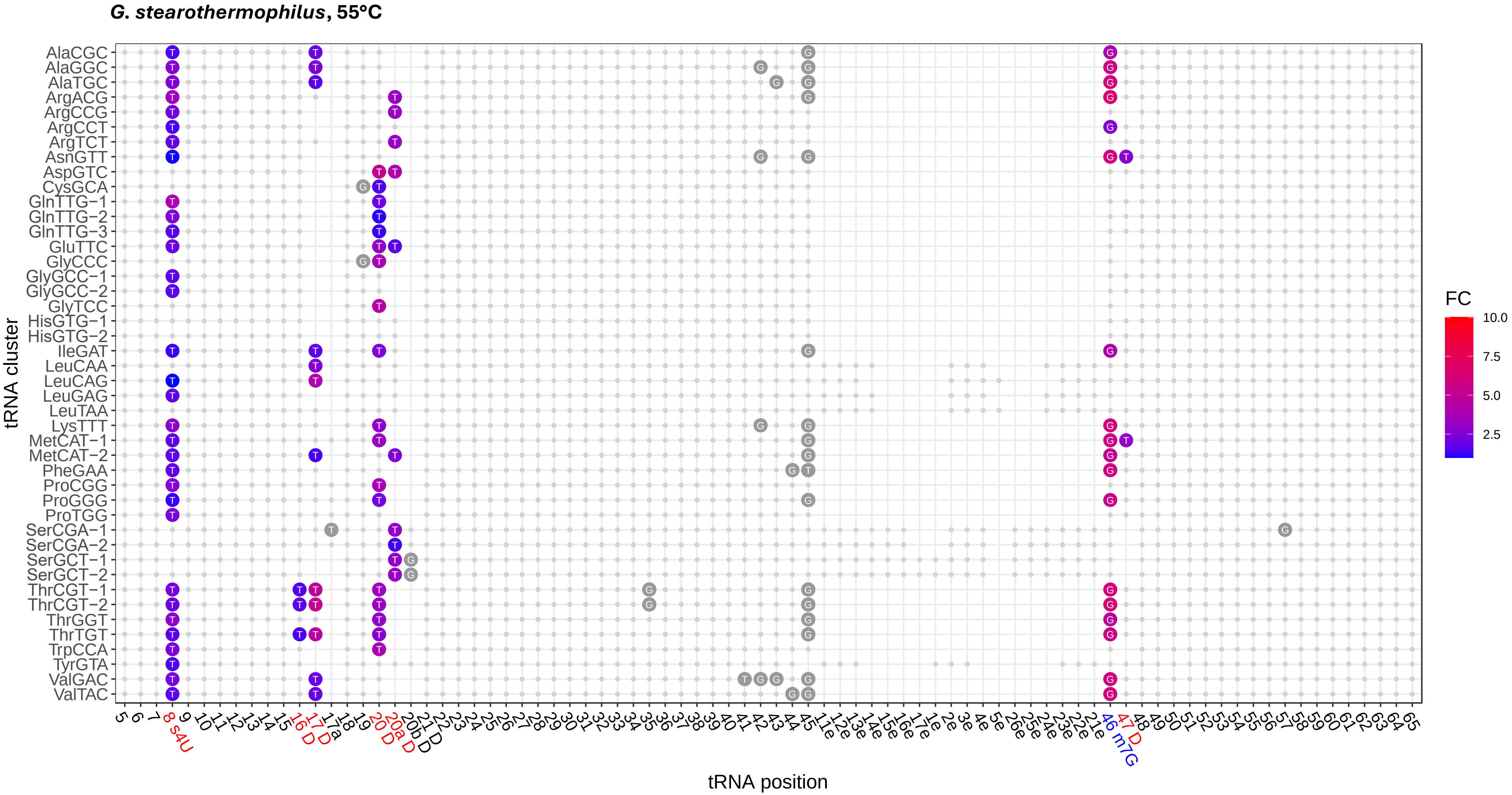
G. stearothermophilus, 55°C
tRNA cluster
AlaCGC
AlaGGC
AlaTGC
ArgACG
ArgCCG
ArgCCT
ArgTCT
AsnGTT
AspGTC
CysGCA
GlnTTG−1
GlnTTG−2
GlnTTG−3
GluTTC
GlyCCC
GlyGCC−1
GlyGCC−2
GlyTCC
HisGTG−1
HisGTG−2
IleGAT
LeuCAA
LeuCAG
LeuGAG
LeuTAA
LysTTT
MetCAT−1
MetCAT−2
PheGAA
ProCGG
ProGGG
ProTGG
SerCGA−1
SerCGA−2
SerGCT−1
SerGCT−2
ThrCGT−1
ThrCGT−2
ThrGGT
ThrTGT
TrpCCA
TyrGTA
ValGAC
ValTAC
tRNA position
8 s4U
16 D
17 D
20 D
20a D
20b D
46 m7G
47 D
FC
10.0
7.5
5.0
2.5

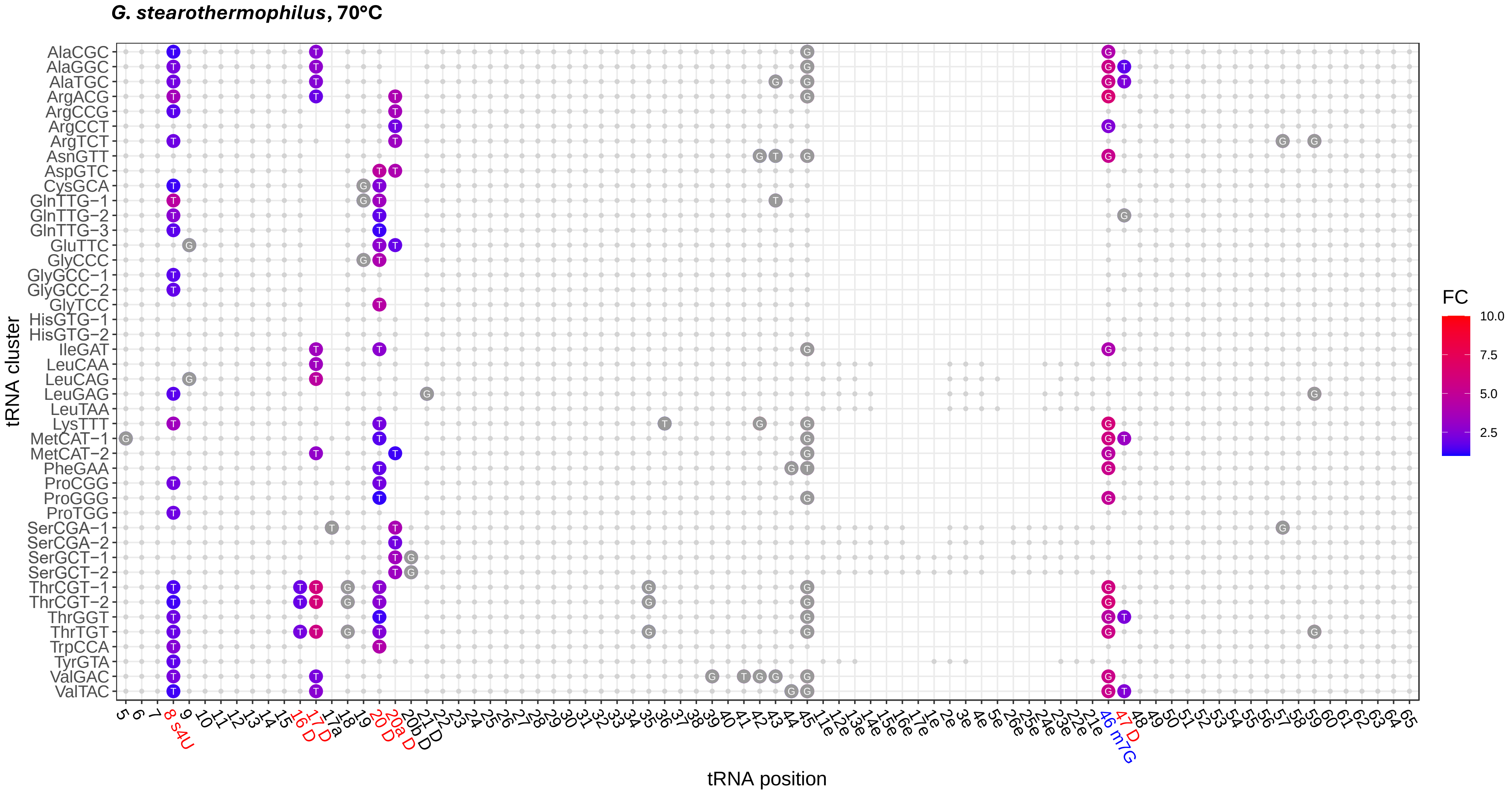

Supplementary Figure S6. Read termination sites from $NaBH_4$-treated RNA seq data of *G. stearothermophilus*. The figure illustrates the investigated RT sites for each tRNA cluster and tRNA position of *G. stearothermophilus* at each growth temperature studied. All tRNA positions exhibiting a significant (adj. P value < 0.01) and strong (FC ≥ 1, total number of RTs ≥ 20, and percentage of RTs ≥ 2) RT sites are color-coded from blue to red based on the logarithmic FC if the RT sites are classified as true positives. Type I false positive points are colored in gray. tRNA sites with no RT enrichment are represented as smaller gray dots. Enhanced RT sites were identified by comparing the RNA seq mapping profiles of $NaBH_4$-treated samples with those of untreated control samples.

***P. halocryophilus*, 10°C**

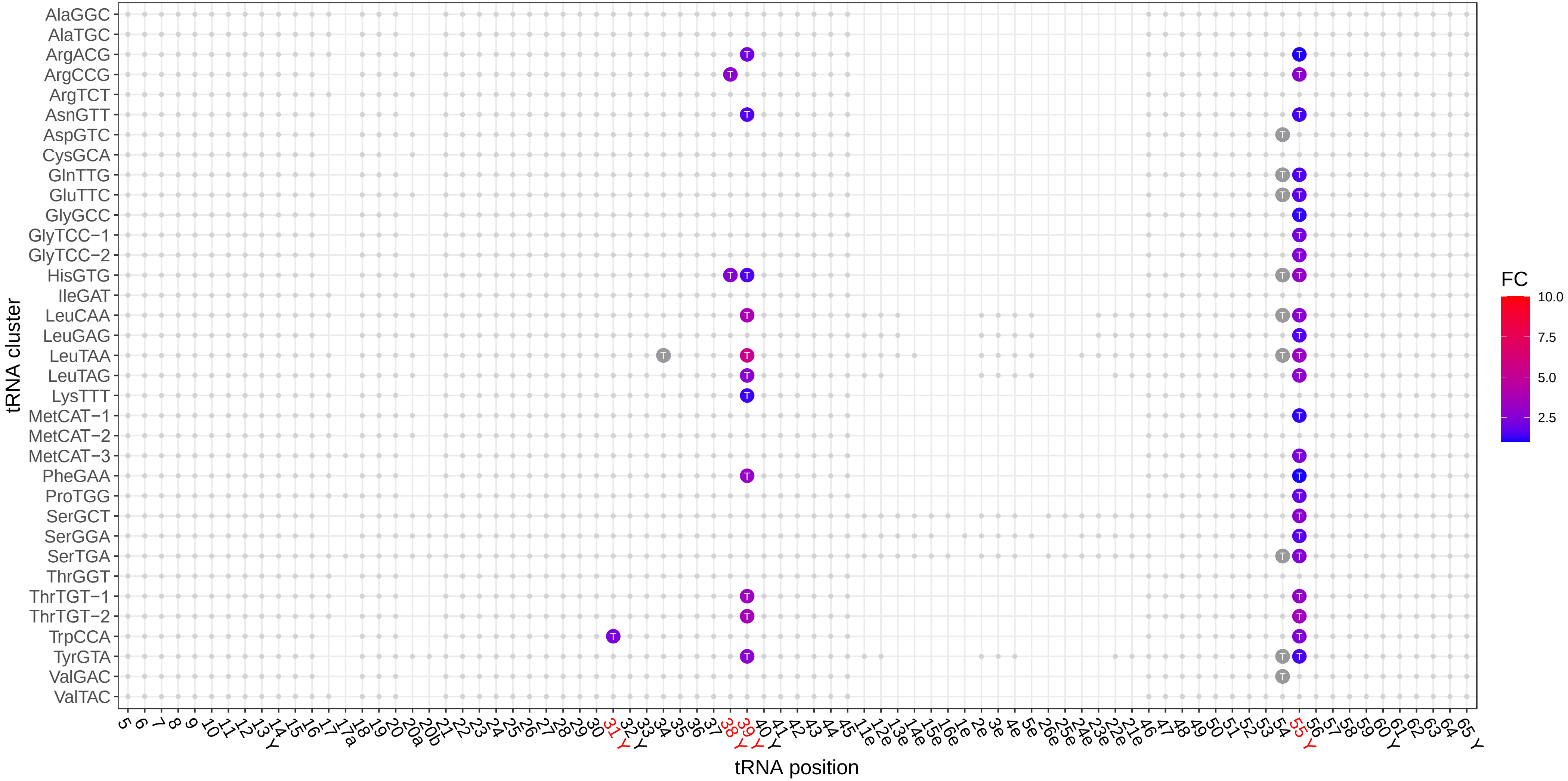

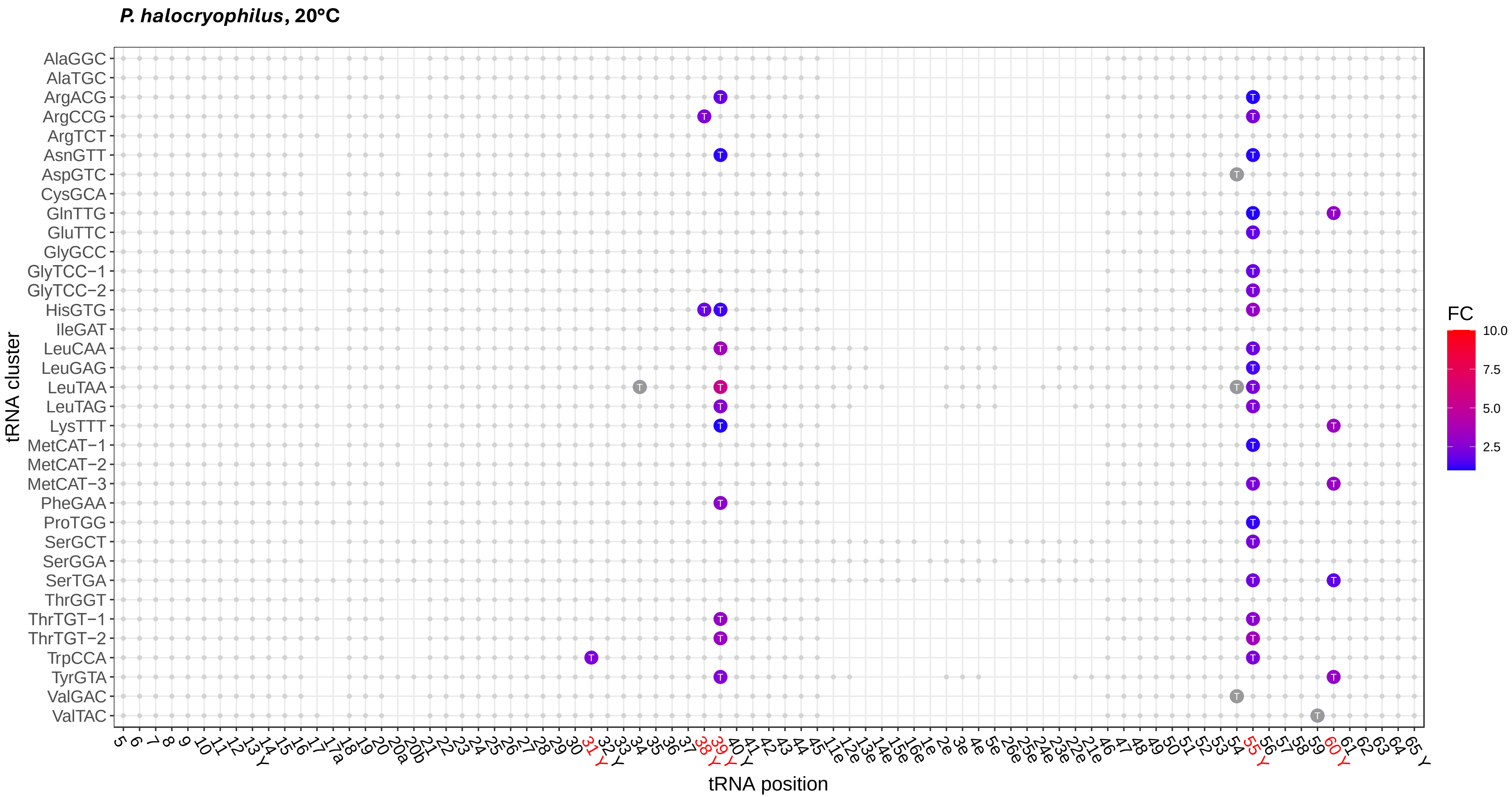

P. halocryophilus, 20°C
tRNA cluster
AlaGGC
AlaTGC
ArgACG
ArgCCG
ArgTCT
AsnGTT
AspGTC
CysGCA
GlnTTG
GluTTC
GlyGCC
GlyTCC−1
GlyTCC−2
HisGTG
IleGAT
LeuCAA
LeuGAG
LeuTAA
LeuTAG
LysTTT
MetCAT−1
MetCAT−2
MetCAT−3
PheGAA
ProTGG
SerGCT
SerGGA
SerTGA
ThrGGT
ThrTGT−1
ThrTGT−2
TrpCCA
TyrGTA
ValGAC
ValTAC
tRNA position
FC
10.0
7.5
5.0
2.5

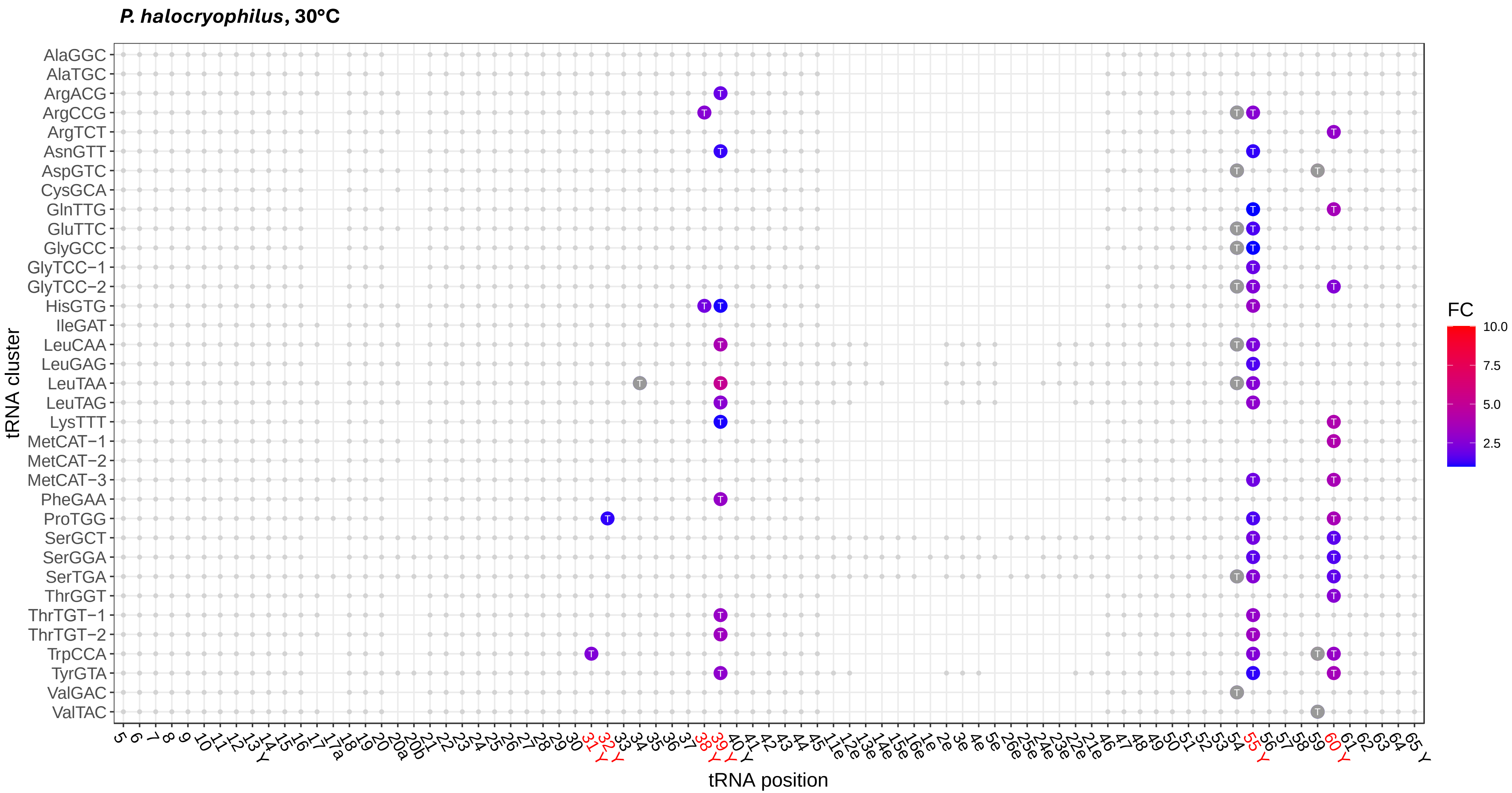

Supplementary Figure S7. Read termination sites from CMCT-treated RNA seq data of *P. halocryophilus*. The figure illustrates the investigated RT sites for each tRNA cluster and tRNA position of *P. halocryophilus* at each growth temperature studied. All tRNA positions exhibiting a significant (adj. P value < 0.01) and strong (FC ≥ 1, total number of RTs ≥ 20, and percentage of RTs ≥ 3) RT sites are color-coded from blue to red based on the logarithmic FC if the RT sites are classified as true positives. Type I false positive points are colored in gray. tRNA sites with no RT enrichment are represented as smaller gray dots. Enhanced RT sites were identified by comparing the RNA seq mapping profiles of 1-cyclohexyl-(2-morpholinoethyl)carbodiimide metho-p-toluene sulfonate (CMCT)-treated samples with those of untreated control samples.

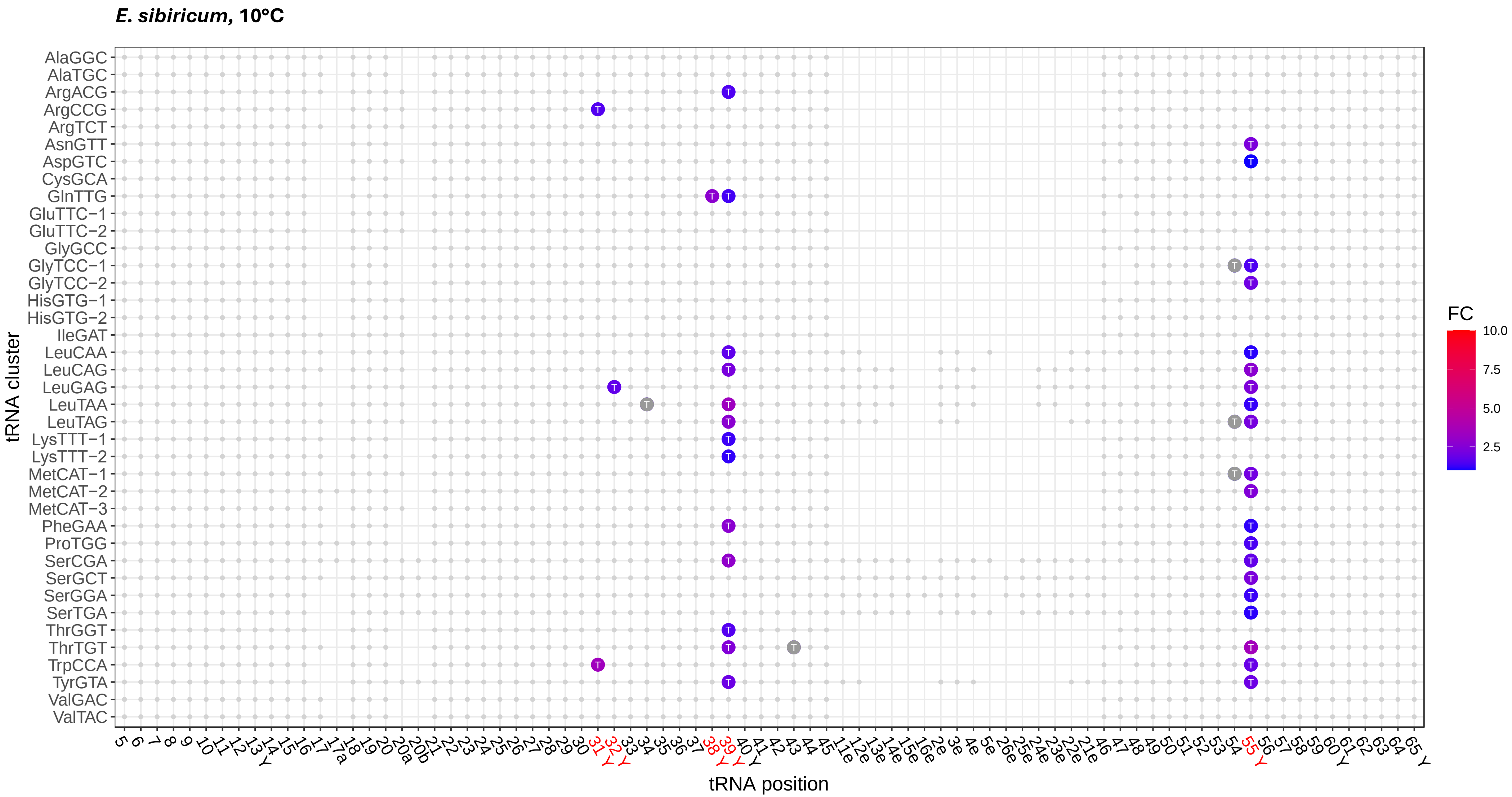

E. sibiricum, 10°C
tRNA cluster
AlaGGC
AlaTGC
ArgACG
ArgCCG
ArgTCT
AsnGTT
AspGTC
CysGCA
GlnTTG
GluTTC−1
GluTTC−2
GlyGCC
GlyTCC−1
GlyTCC−2
HisGTG−1
HisGTG−2
IleGAT
LeuCAA
LeuCAG
LeuGAG
LeuTAA
LeuTAG
LysTTT−1
LysTTT−2
MetCAT−1
MetCAT−2
MetCAT−3
PheGAA
ProTGG
SerCGA
SerGCT
SerGGA
SerTGA
ThrGGT
ThrTGT
TrpCCA
TyrGTA
ValGAC
ValTAC
tRNA position
FC
10.0
7.5
5.0
2.5

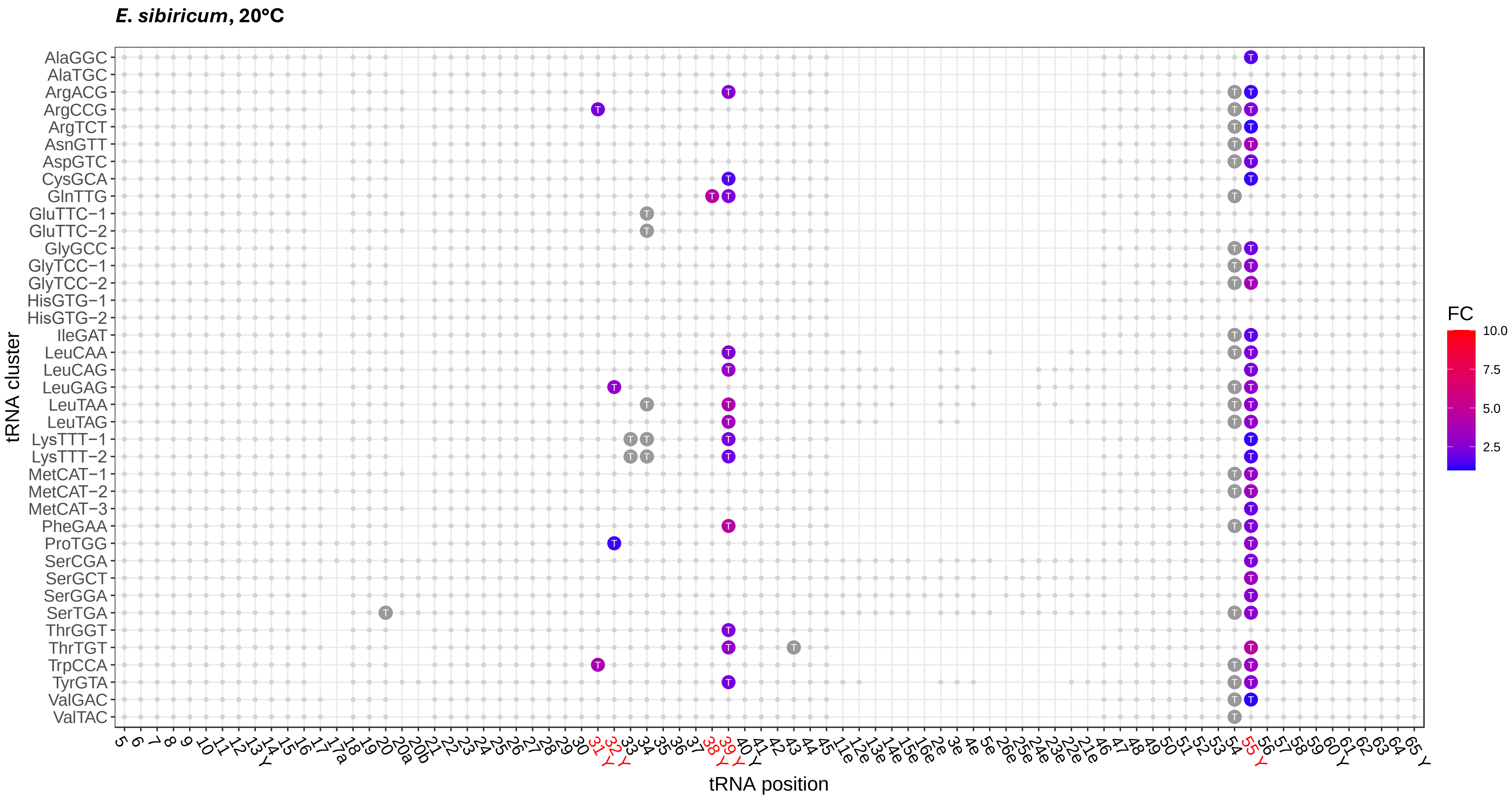
E. sibiricum, 20°C
tRNA cluster
tRNA position
FC
10.0
7.5
5.0
2.5
AlaGGC
AlaTGC
ArgACG
ArgCCG
ArgTCT
AsnGTT
AspGTC
CysGCA
GlnTTG
GluTTC−1
GluTTC−2
GlyGCC
GlyTCC−1
GlyTCC−2
HisGTG−1
HisGTG−2
IleGAT
LeuCAA
LeuCAG
LeuGAG
LeuTAA
LeuTAG
LysTTT−1
LysTTT−2
MetCAT−1
MetCAT−2
MetCAT−3
PheGAA
ProTGG
SerCGA
SerGCT
SerGGA
SerTGA
ThrGGT
ThrTGT
TrpCCA
TyrGTA
ValGAC
ValTAC

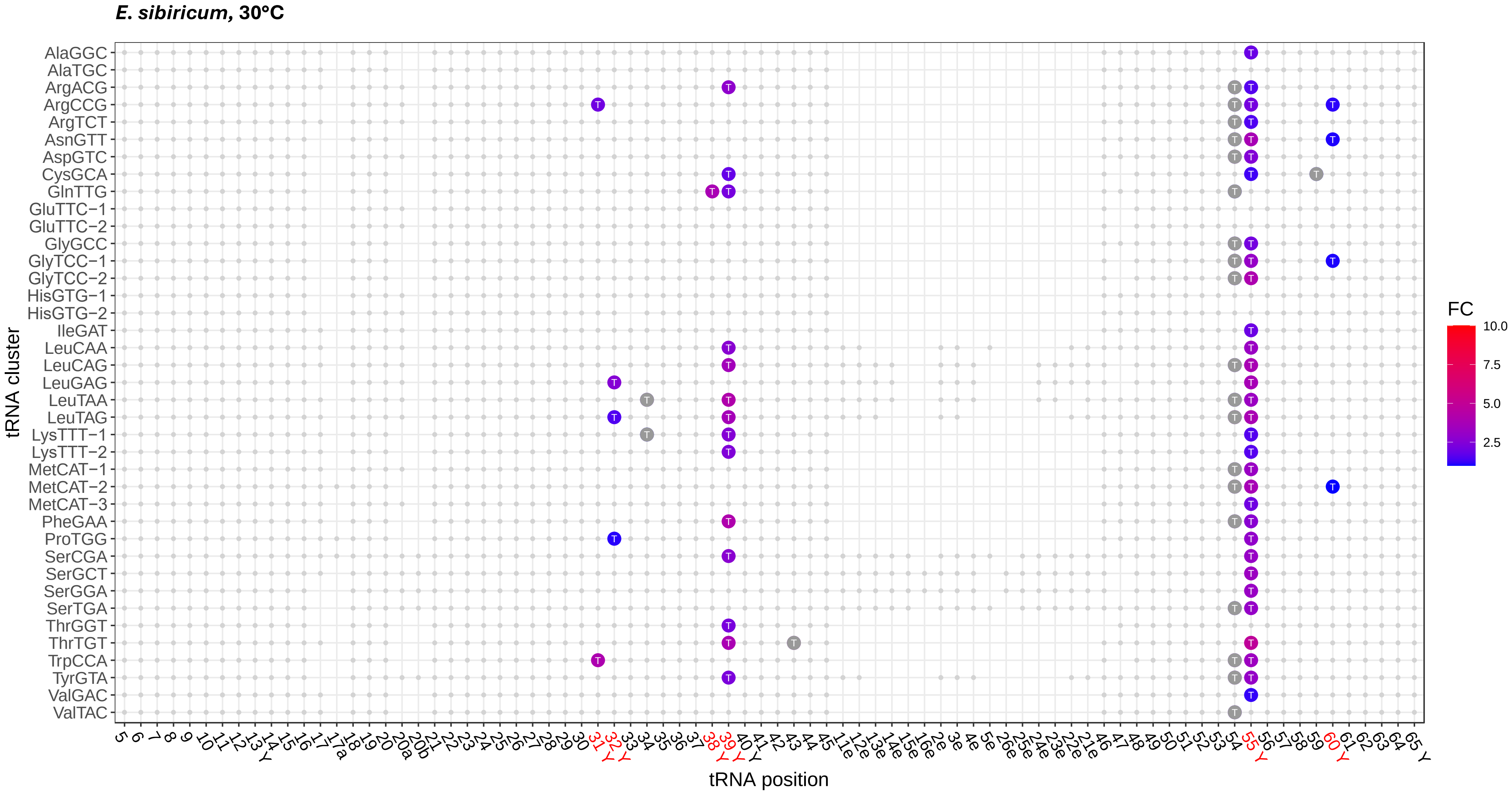

Supplementary Figure S8. Read termination sites from CMCT-treated RNA seq data of *E. sibiricum*. The figure illustrates the investigated RT sites for each tRNA cluster and tRNA position of *E. sibiricum* at each growth temperature studied. All tRNA positions exhibiting a significant (adj. P value < 0.01) and strong (FC ≥ 1, total number of RTs ≥ 20, and percentage of RTs ≥ 3) RT sites are color-coded from blue to red based on the logarithmic FC if the RT sites are classified as true positives. Type I false positive points are colored in gray. tRNA sites with no RT enrichment are represented as smaller gray dots. Enhanced RT sites were identified by comparing the RNA seq mapping profiles of CMCT-treated samples with those of untreated control samples.

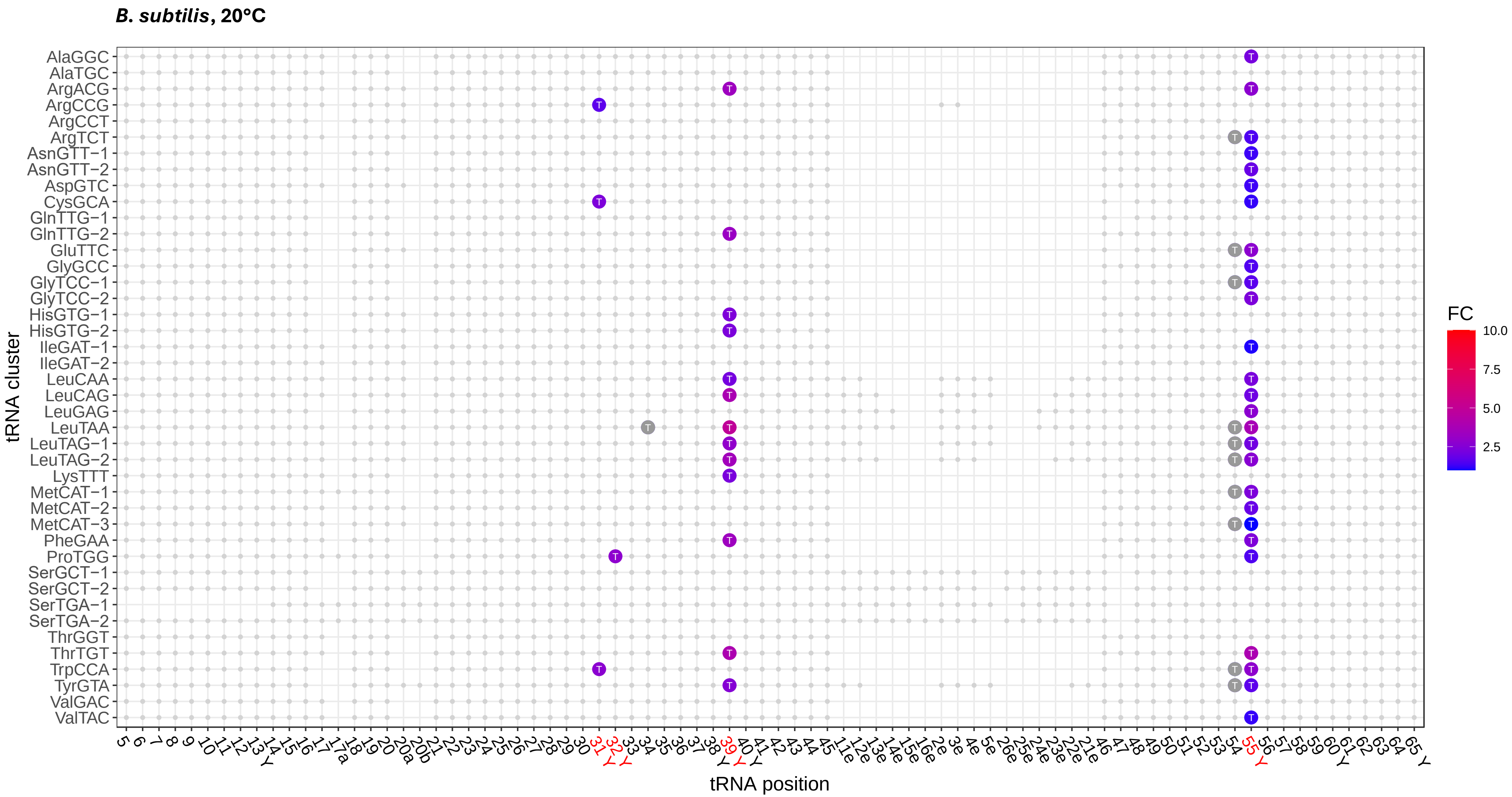
B. subtilis, 20°C
tRNA cluster
AlaGGC
AlaTGC
ArgACG
ArgCCG
ArgCCT
ArgTCT
AsnGTT−1
AsnGTT−2
AspGTC
CysGCA
GlnTTG−1
GlnTTG−2
GluTTC
GlyGCC
GlyTCC−1
GlyTCC−2
HisGTG−1
HisGTG−2
IleGAT−1
IleGAT−2
LeuCAA
LeuCAG
LeuGAG
LeuTAA
LeuTAG−1
LeuTAG−2
LysTTT
MetCAT−1
MetCAT−2
MetCAT−3
PheGAA
ProTGG
SerGCT−1
SerGCT−2
SerTGA−1
SerTGA−2
ThrGGT
ThrTGT
TrpCCA
TyrGTA
ValGAC
ValTAC
tRNA position
FC
10.0
7.5
5.0
2.5

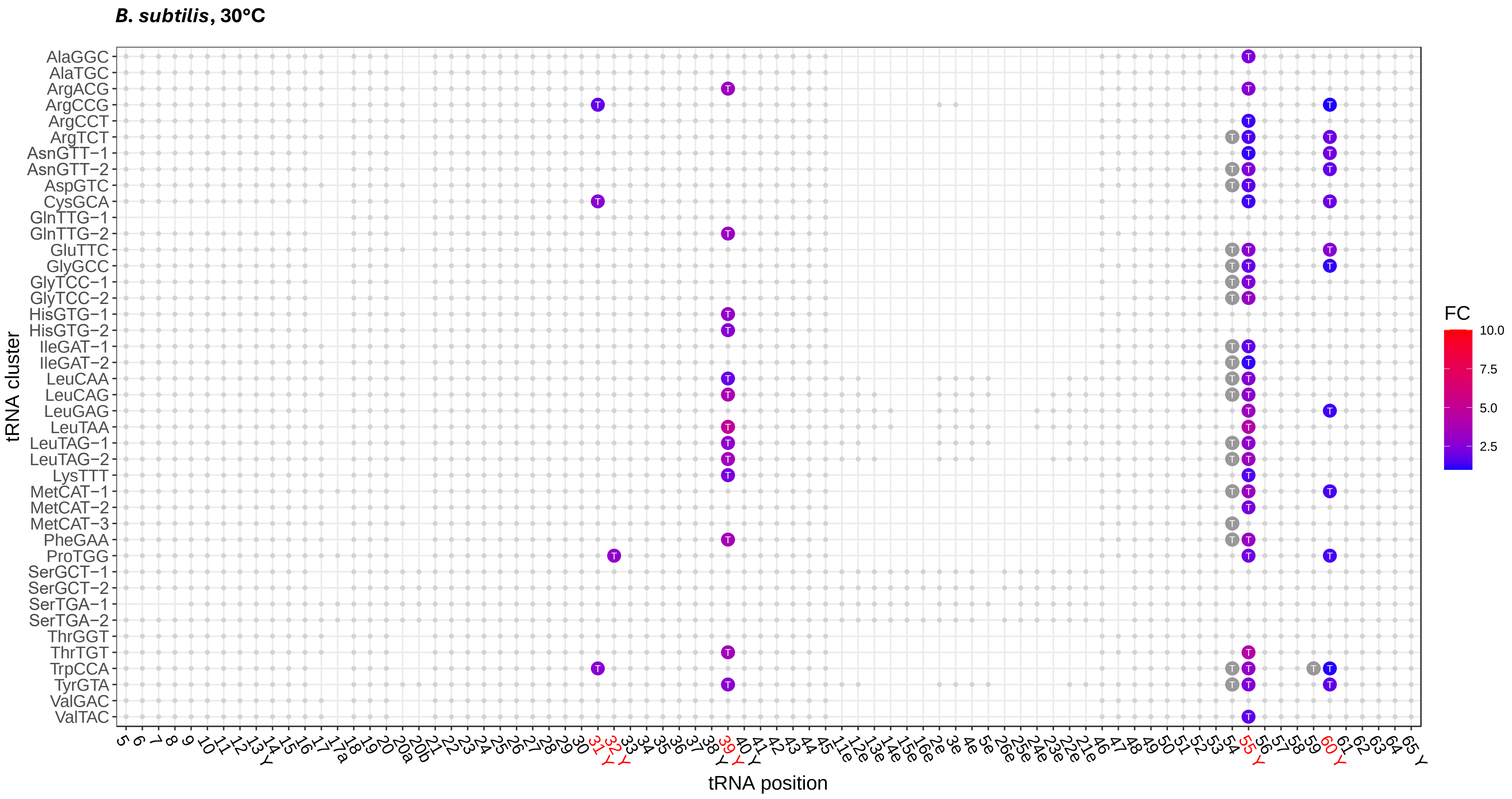

B. subtilis, 30°C
tRNA cluster
AlaGGC
AlaTGC
ArgACG
ArgCCG
ArgCCT
ArgTCT
AsnGTT−1
AsnGTT−2
AspGTC
CysGCA
GlnTTG−1
GlnTTG−2
GluTTC
GlyGCC
GlyTCC−1
GlyTCC−2
HisGTG−1
HisGTG−2
IleGAT−1
IleGAT−2
LeuCAA
LeuCAG
LeuGAG
LeuTAA
LeuTAG−1
LeuTAG−2
LysTTT
MetCAT−1
MetCAT−2
MetCAT−3
PheGAA
ProTGG
SerGCT−1
SerGCT−2
SerTGA−1
SerTGA−2
ThrGGT
ThrTGT
TrpCCA
TyrGTA
ValGAC
ValTAC
5 6 7 8 9 10 11 12 13 14 15 16 17 17a 18 19 20 20a 20b 21 22 23 24 25 26 27 28 29 30 31 32 33 34 35 36 37 38 39 40 41 42 43 44 45 11e 12e 13e 14e 15e 16e 2e 3e 4e 5e 26e 25e 24e 23e 22e 21e 46 47 48 49 50 51 52 53 54 55 56 57 58 59 60 61 62 63 64 65
tRNA position
FC
10.0
7.5
5.0
2.5

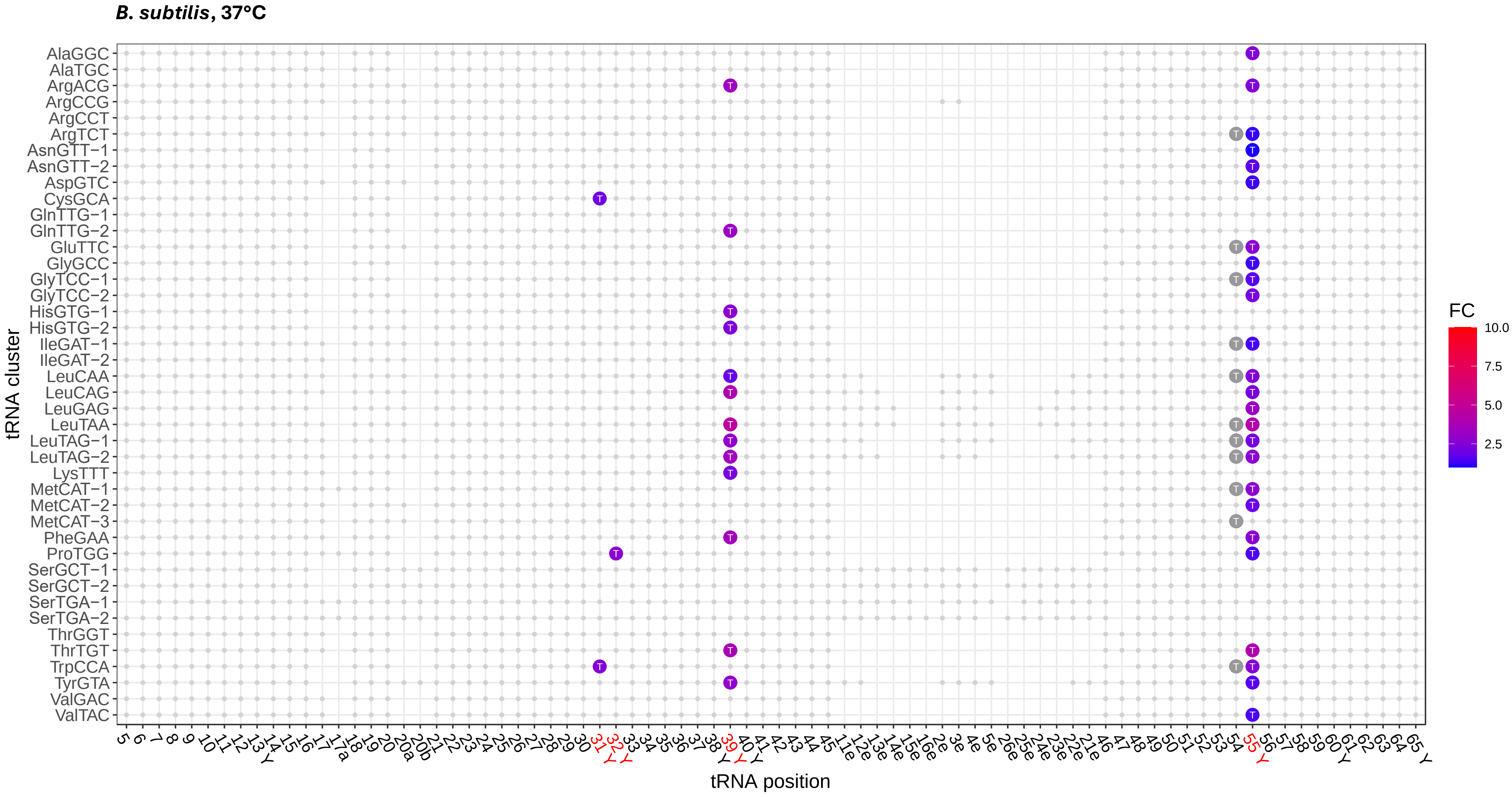

Supplementary Figure S9. Read termination sites from CMCT-treated RNA seq data of *B. subtilis*. The figure illustrates the investigated RT sites for each tRNA cluster and tRNA position of *B. subtilis* at each growth temperature studied. All tRNA positions exhibiting a significant (adj. P value < 0.01) and strong (FC ≥ 1, total number of RTs ≥ 20, and percentage of RTs ≥ 3) RT sites are color-coded from blue to red based on the logarithmic FC if the RT sites are classified as true positives. Type I false positive points are colored in gray. tRNA sites with no RT enrichment are represented as smaller gray dots. Enhanced RT sites were identified by comparing the RNA seq mapping profiles of CMCT-treated samples with those of untreated control samples.

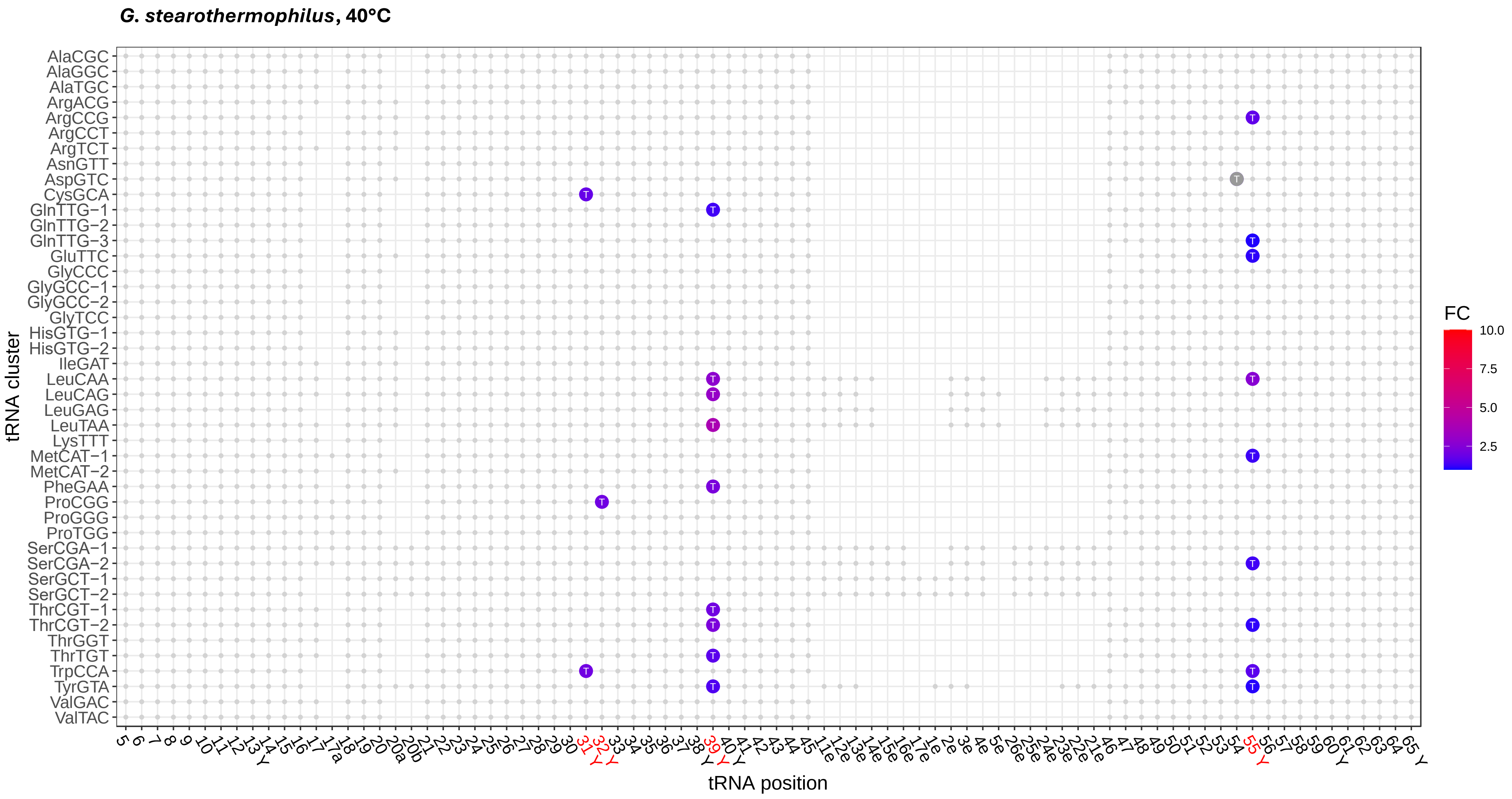
G. stearothermophilus, 40°C
AlaCGC
AlaGGC
AlaTGC
ArgACG
ArgCCG
ArgCCT
ArgTCT
AsnGTT
AspGTC
CysGCA
GlnTTG−1
GlnTTG−2
GlnTTG−3
GluTTC
GlyCCC
GlyGCC−1
GlyGCC−2
GlyTCC
HisGTG−1
HisGTG−2
IleGAT
LeuCAA
LeuCAG
LeuGAG
LeuTAA
LysTTT
MetCAT−1
MetCAT−2
PheGAA
ProCGG
ProGGG
ProTGG
SerCGA−1
SerCGA−2
SerGCT−1
SerGCT−2
ThrCGT−1
ThrCGT−2
ThrGGT
ThrTGT
TrpCCA
TyrGTA
ValGAC
ValTAC
tRNA cluster
tRNA position
FC
10.0
7.5
5.0
2.5

Gst

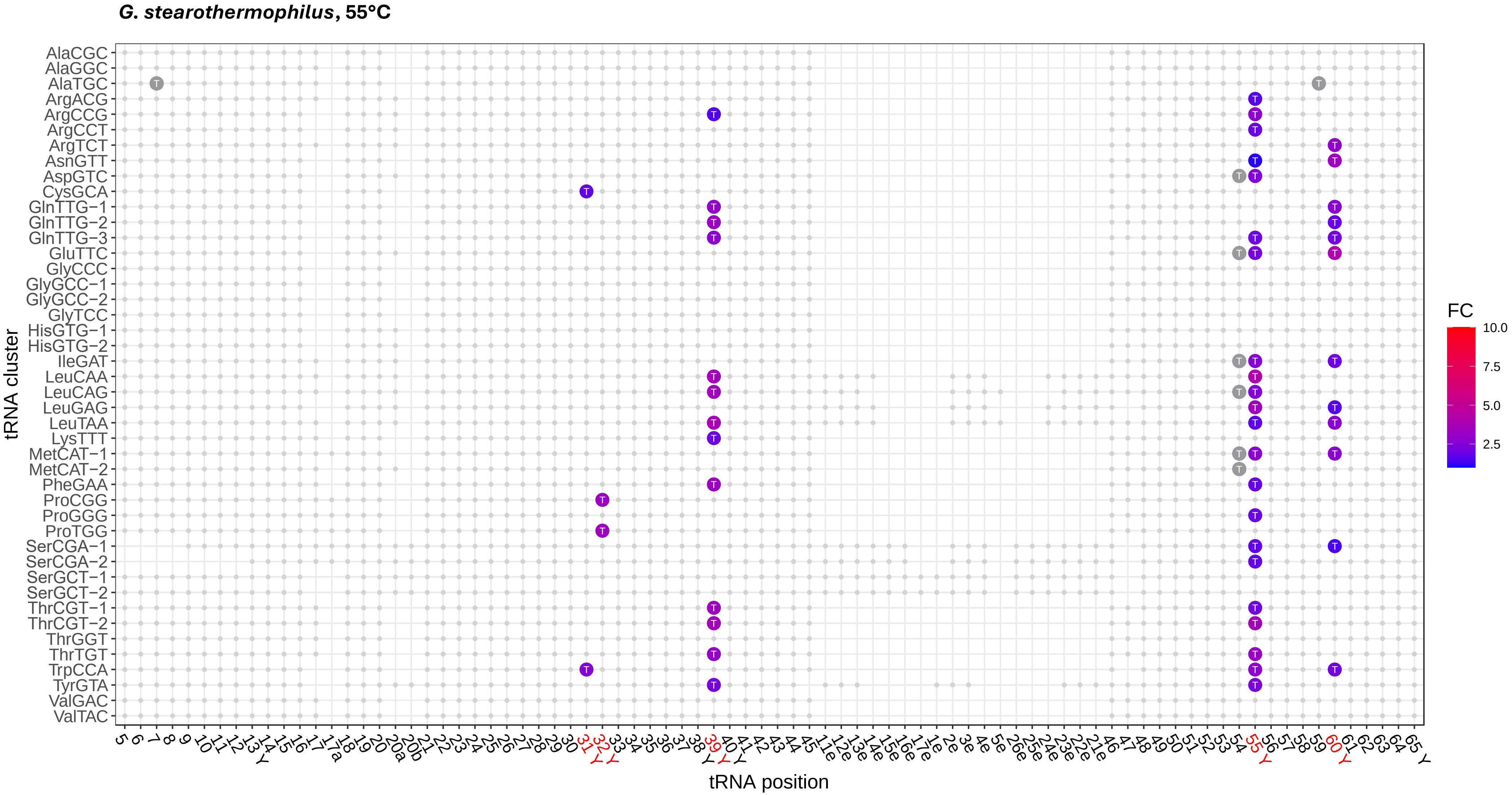
G. stearothermophilus, 55°C
tRNA cluster
AlaCGC
AlaGGC
AlaTGC
ArgACG
ArgCCG
ArgCCT
ArgTCT
AsnGTT
AspGTC
CysGCA
GlnTTG−1
GlnTTG−2
GlnTTG−3
GluTTC
GlyCCC
GlyGCC−1
GlyGCC−2
GlyTCC
HisGTG−1
HisGTG−2
IleGAT
LeuCAA
LeuCAG
LeuGAG
LeuTAA
LysTTT
MetCAT−1
MetCAT−2
PheGAA
ProCGG
ProGGG
ProTGG
SerCGA−1
SerCGA−2
SerGCT−1
SerGCT−2
ThrCGT−1
ThrCGT−2
ThrGGT
ThrTGT
TrpCCA
TyrGTA
ValGAC
ValTAC
tRNA position
FC
10.0
7.5
5.0
2.5

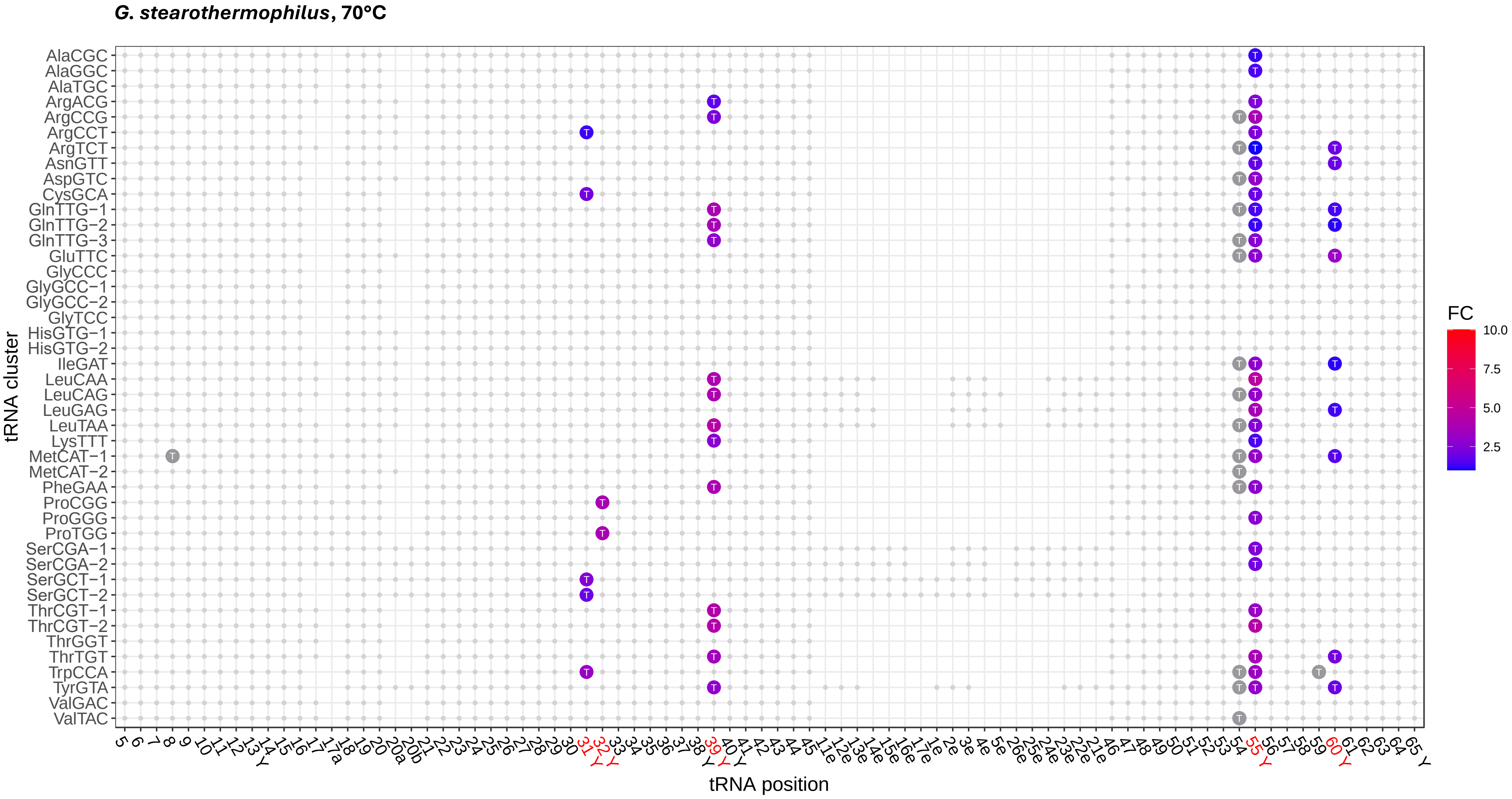

Supplementary Figure 10. Read termination sites from CMCT-treated RNA seq data of *G. stearothermophilus*. The figure illustrates the investigated RT sites for each tRNA cluster and tRNA position of *G. stearothermophilus* at each growth temperature studied. All tRNA positions exhibiting a significant (adj. P value < 0.01) and strong (FC ≥ 1, total number of RTs ≥ 20, and percentage of RTs ≥ 3) RT sites are color-coded from blue to red based on the logarithmic FC if the RT sites are classified as true positives. Type I false positive points are colored in gray. tRNA sites with no RT enrichment are represented as smaller gray dots. Enhanced RT sites were identified by comparing the RNA seq mapping profiles of CMCT-treated samples with those of untreated control samples.

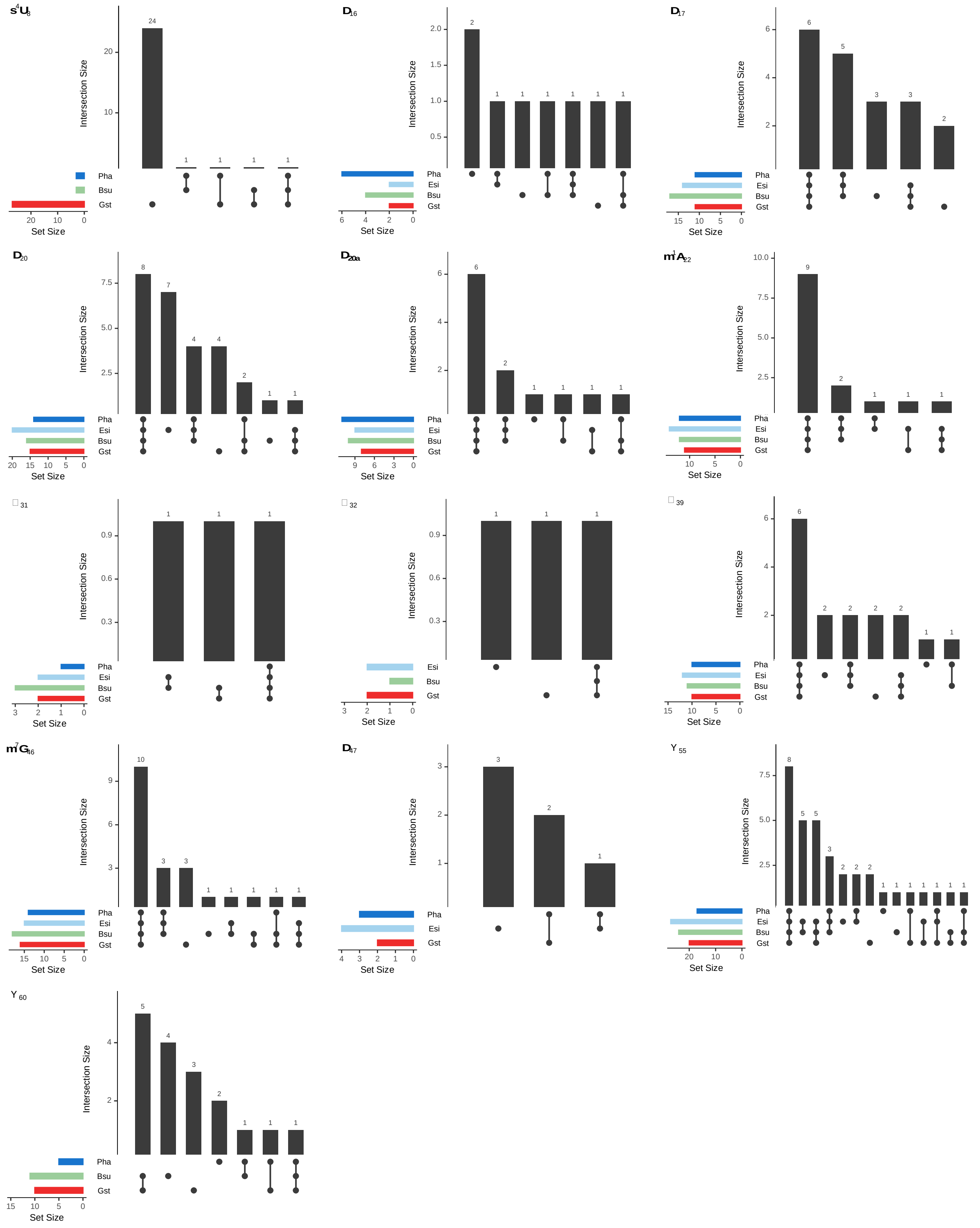

Supplementary Figure S11. Overlap in the numbers of modified tRNA families across different bacteria. Upset plots depict the intersection of modified tRNA families categorized by their encoded amino acids and their respective anticodons (unique counts). The counts are derived from RNA sequencing data obtained at the optimal growth temperatures of the cultured bacteria, which are 20°C for P. halocryophilus and E. sibiricum, 30°C for B. subtilis, and 55°C for G. stearothermophilus.

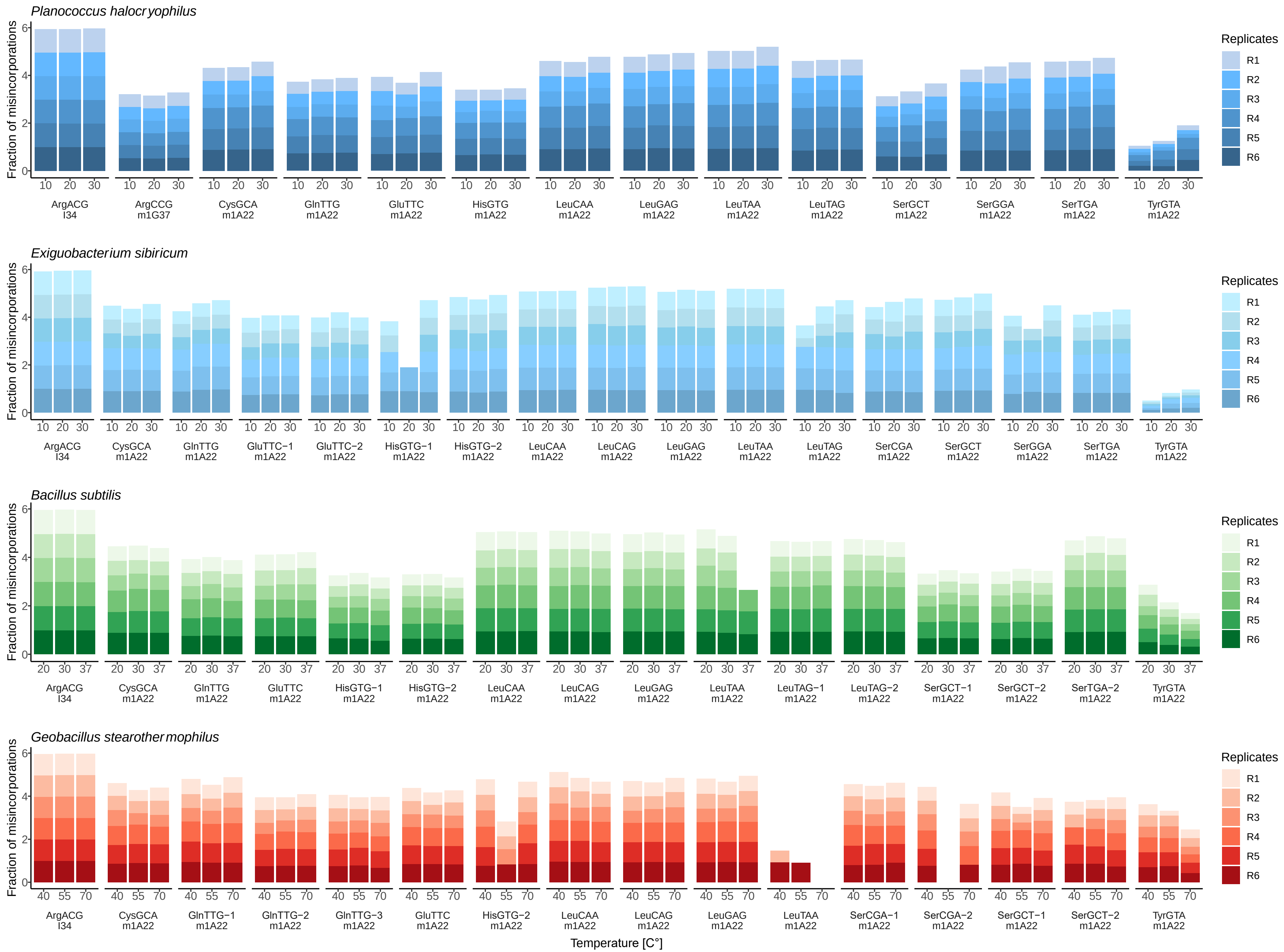

Planococcus halocryophilus
Exiguobacterium sibiricum
Bacillus subtilis
Geobacillus stearothermophilus
Fraction of misincorporations
Replicates
R1
R2
R3
R4
R5
R6
ArgACG I34
ArgCCG m1G37
CysGCA m1A22
GlnTTG m1A22
GluTTC m1A22
HisGTG m1A22
LeuCAA m1A22
LeuGAG m1A22
LeuTAA m1A22
LeuTAG m1A22
SerGCT m1A22
SerGGA m1A22
SerTGA m1A22
TyrGTA m1A22
GluTTC−1 m1A22
GluTTC−2 m1A22
HisGTG−1 m1A22
HisGTG−2 m1A22
LeuCAG m1A22
SerCGA m1A22
LeuTAG−1 m1A22
LeuTAG−2 m1A22
SerGCT−1 m1A22
SerGCT−2 m1A22
SerTGA−2 m1A22
GlnTTG−1 m1A22
GlnTTG−2 m1A22
GlnTTG−3 m1A22
SerCGA−1 m1A22
SerCGA−2 m1A22
Temperature [C°]

Supplementary Figure S12. Fraction of base mismatches for m^1A, I and m^1G tRNA modifications. The fraction of misincorporations [nucleotide mismatches / original nucleotide] in the mapping profile at positions 22, 34, and 37 are shown for each affected tRNA, as well as the investigated bacteria and temperatures. The proportion of misincorporations is given for each biological replicate [R1-R6] and these were only considered if more than 20 reads were available. Inosine (I)34 and 1-methyladenosine (m^1A)22 were identified in each bacterium, while 1-methylguanosine (m^1G)37 was only found at $tRNA^{Arg}_{CCG}$ of *P. halocryophilus*.
